# Prognostic Impact of *ERAP1* Genetic Variants and Haplotypes in Cervical Cancer: Insights From TCGA‐CESC and a Brazilian Patient Cohort

**DOI:** 10.1002/mgg3.70170

**Published:** 2026-10-20

**Authors:** Ana Luisa Dian, Ana Paula Almeida Cunha, Carolina Pereira de Souza Melo, Maria do Desterro Soares Brandão Nascimento, Maria Gabriela Reis Carvalho, Jeronimo Conceição Ruiz

**Affiliations:** ^1^ Grupo Informática de Biossistemas, Bioengenharia e Genômica, Instituto René Rachou, Fiocruz Minas Belo Horizonte Brazil; ^2^ Programa de Pós‐graduação Stricto sensu em Biologia Computacional e Sistemas do Instituto Oswaldo Cruz, IOC, Fiocruz Rio de Janeiro Brazil; ^3^ Department of Pathology Universidade Federal Do Maranhão ‐ UFMA São Luís Brazil

**Keywords:** cervical cancer, *ERAP1* genetic variants, prognostic biomarkers, survival analysis

## Abstract

**Background:**

This study aimed to evaluate the association between genetic variants and haplotypes of *ERAP1* and clinical outcomes in cervical cancer patients, utilizing data from the TCGA‐CESC and a cohort of Brazilian patients.

**Methods:**

*ERAP1* variants and haplotypes were evaluated in two distinct cohorts: whole‐exome sequencing (WES) data from 173 cervical cancer cases in the TCGA‐CESC dataset, and Sanger sequencing data from 52 Brazilian cervical cancer patients. The TCGA‐CESC data were employed for *in silico* analysis to identify significant *ERAP1* variants and haplotypes, which were subsequently evaluated in Brazilian cervical cancer samples. The Tumor IMmune Estimation Resource (TIMER) tool was used to assess the association between *ERAP1* expression and immune cell infiltration.

**Results:**

Haplotype analysis of *ERAP1* in the TCGA‐CESC dataset revealed that H005 (rs26653—rs2287987—rs30187—rs10050860—rs17482078—rs27044) and H023 (rs26653—rs27895—rs30187—rs27044) haplotypes were significantly associated with poorer survival outcomes. Homozygosity for H005 and the presence of H023 were strongly associated with both overall survival (OS) (HR = 9.591, *p* = 0.00384; HR = 10.183, *p* = 0.02862, respectively) and disease‐specific survival (DSS) (HR = 12.851, *p* = 0.00162; HR = 15.183, *p* = 0.01191, respectively). TCGA‐CESC data showed that homozygosity for rs2287987 (p.Met349Val) was associated with an increased risk of death in both overall survival (OS) (HR = 11.043, *p* = 0.00376) and disease‐specific survival (DSS) (HR = 13.833, *p* = 0.00255), whereas rs30187 (p.Lys528Arg) demonstrated a protective effect for DSS, reducing mortality risk in both heterozygous (HR = 0.374, *p* = 0.04055) and homozygous (HR = 0.346, *p* = 0.03845) forms. However, although these variants were individually associated with survival outcomes in the dataset, this association was not retained in the multivariate analysis, suggesting that these variants may not have an independent prognostic effect. Similarly, no associations were found for these variants in the Brazilian cervical cancer cohort, suggesting that the impact of these variants on the prognosis of cervical cancer patients may be primarily influenced by the combination of variants. Furthermore, *ERAP1* expression levels were positively correlated with CD8^+^ T cell infiltration (correlation coefficient = 0.264, *p* = 0.00000960), which was associated with improved survival outcomes (HR = 0.006, *p* = 0.049).

**Conclusion:**

*ERAP1* haplotype H005 homozygosity and the presence of H023 were linked to poorer prognosis, indicating their potential as prognostic biomarkers in cervical cancer patients. Individually, the rs2287987 variant showed a potential association with worse outcomes in the TCGA dataset; however, this finding was not replicated among Brazilian patients, suggesting the potential role of these variants when considered as haplotypes in the function of *ERAP1*. Additionally, the protective role of CD8+ T cell infiltration highlights its relevance as a possible therapeutic target for improving survival in cervical cancer patients.

## Graphical abstract

Overview of the study design and main findings on the prognostic impact of ERAP1 genetic variants, haplotypes, and expression in cervical cancer. Genetic and clinical data from TCGA‐CESC and a Brazilian patient cohort were analyzed to evaluate associations with survival outcomes. Specific ERAP1 variants and haplotypes were associated with either reduced mortality risk (rs30187) or increased risk of death (rs2287987, H005, and H023) in the TCGA cohort, whereas no significant associations were observed in Brazilian patients. Additionally, ERAP1 expression was positively correlated with immune cell infiltration, particularly CD8^+^ T cells, and higher CD8+ T‐cell infiltration was associated with improved survival, highlighting the role of ERAP1‐mediated antigen processing in antitumor immune responses in cervical cancer.

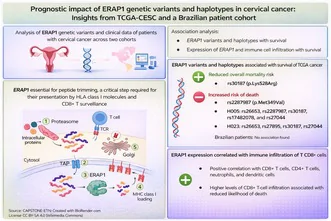

**Abbreviations**
AbraOM: Online Archive of Brazilian Mutations
APM: antigen processing machinery
CI: confidence interval
CRC: colorectal cancer
CTL: cytotoxic T‐lymphocyte
DSS: disease‐specific survival
*ERAP1*
: endoplasmic reticulum aminopeptidase
FIGO: International Federation of Gynecology and Obstetrics
GWAS: genome‐wide association studies
HLA‐I: human leukocyte antigen
HPV: human papillomavirus
HR: hazard ratio
MAF: minor allele frequency
mRNA: messenger ribonucleic acid
NCI: National Cancer Institute
NSCLC: non‐small cell lung carcinoma
OPSCC: oropharyngeal squamous cell carcinoma
OS: overall survival
TCGA‐CESC: cervical squamous cell carcinoma and endocervical adenocarcinoma in The Cancer Genome Atlas
WES: whole exome sequencing

## 1 Introduction

Cervical cancer remains a prevalent malignant disease that affects women worldwide, especially in developing countries, despite the implementation of screening and vaccination initiatives. In 2020, an estimated 604,000 new cases and 342,000 deaths associated with the disease were reported, representing 6.5% of all cancers in women (Sung et al. 2021). Approximately 90% of cervical cancer‐associated deaths occur in low‐ and middle‐income countries due to limited access to cancer screening and effective therapeutic options (Sung et al. 2021). In Brazil, 6627 deaths were reported in 2020, which represents a mortality rate of 6.12 deaths per 100,000 women (INCA 2023). The National Cancer Institute (NCI) estimated that 17,010 new cases of cervical cancer annually worldwide will be diagnosed annualy between 2023 and 2025, resulting in 6570 deaths (INCA 2023).

Persistent *human papillomavirus* (HPV) infection is considered the primary risk factor for the development of cervical cancer. Several mechanisms that allow HPV‐infected and tumor cells to evade immune surveillance and overcome host immune control have been identified (Steinbach and Riemer 2018). One such mechanism involves insufficient processing and presentation of antigens on the cell surface by *human leukocyte antigen class I* (*HLA‐I*) complexes, which can directly impede the generation of effective cytotoxic T lymphocytes (CTL) epitopes (Steinbach and Riemer 2018; Saulle et al. 2020).

The process by which the human cellular immune system identifies damaged or infected cells involves the presentation of peptides derived from the proteolytic processing of intracellular proteins. The correct processing of these endogenous proteins by the antigen processing machinery (APM), and the subsequent presentation of peptides on the cell surface by *HLA‐I* molecules are crucial for the activation of adaptive and antitumor immune responses mediated by cytotoxic T lymphocytes (CD8^+^) (Mehta et al. 2009). Peptides, whether immunogenic or not, are initially generated through a series of proteolytic events by the proteasome complex in the cytoplasm, which cleaves intracellular proteins into heterogeneous fragments (Saulle et al. 2020). Peptides are then transported to the endoplasmic reticulum (ER) where they undergo further processing by the transporter associated with antigen processing (*TAP*). In the ER, the peptides are subjected to N‐terminal trimming by the endoplasmic reticulum aminopeptidase 1 (*ERAP1*) to produce peptides of the appropriate length for binding to HLA‐I molecules (Hewitt 2003). *ERAP1* plays a crucial role in shaping conformationally stable peptides suited to be loaded onto *HLA‐I* molecules. It preferentially cleaves peptides with large hydrophobic C‐terminal amino acids and exhibits a strong tendency to trim peptides of 9–16 into 8–9 amino acids, which is the required length for binding to HLA‐I molecules (Hewitt 2003; Jhunjhunwala et al. 2021; Kelly and Trowsdale 2019; Saric et al. 2002).

*The ERAP1* gene, located on chromosome 5q15, spans 47,379bp in length and consists of 20 exons (Jhunjhunwala et al. 2021). It is a naturally polymorphic gene, and its genetic variability contributes to predisposition to diseases caused by failures in the adaptive immune response (Steinbach and Riemer 2018; Stratikos et al. 2014). GWAS analyses have identified *ERAP1* variants associated with autoimmune diseases, such as ankylosing spondylitis, as well as different types of cancer (Stratikos et al. 2014; Guerini et al. 2012; Reeves et al. 2013, 2019). These associations often occur in conjunction with *HLA‐I* alleles, suggesting a direct impact on antigen processing and tumor antigen presentation to the immune system (Stratikos et al. 2014). *ERAP1* genetic variants are primarily located near the catalytic site (AA residues 346–349), within the peptide‐binding site (AA residues 725 and 730), and within structural domains that regulate its conformational transitions (AA residues 528–575). Alterations in these regions reduce the enzymatic efficiency of processing antigenic peptides, impairing the presentation of tumor epitopes to CD8+ T lymphocytes, which may facilitate immune evasion of malignant cells. Among the variants are rs2287987 (p.Met349Val), rs30187 (p.Lys528Arg), rs10050860 (p.Asp575Asn), rs17482078 (p.Arg725Gln), and rs27044 (p.Gln730Glu), which showed correlation with modifications in peptide length preference and trimming specificity (Saulle et al. 2020; Reeves et al. 2019, 2020). Studies have demonstrated that *ERAP1* variants, when combined into haplotypes, can have a synergistic impact, amplifying their effects on peptide trimming activity (Mehta et al. 2009, 2008, 2015; Reeves et al. 2013, 2019; Padula et al. 2020; Wu and Zhao 2021). Reeves et al. (2013) identified 13 *ERAP1* haplotypes, classified according to their ability to generate appropriately sized antigenic epitopes. The haplotype encompassing rs2287987 (p.Met349Val), rs30187 (p.Lys528Arg), rs10050860 (p.Asp575Asn), rs17482078 (p.Arg725Gln), and rs27044 (p.Gln730Glu) variants was found to encode a nonfunctional protein, with each variant independently associated with a series of diseases or susceptibility. Additionally, *ERAP1* variants were previously reported as associated with several cancer types, including colorectal cancer (Zou et al. 2024), non‐small cell lung cancer (Wiśniewski et al. 2021; Yang et al. 2021), neuroblastoma (Tempora et al. 2024), oropharyngeal squamous cell carcinomas (Reeves et al. 2019, 2020), and cervical cancer (Mehta et al. 2015, 2017). Since proper antigen presentation is crucial for immune activation, individuals carrying specific *ERAP1* haplotypes in combination with particular HLA‐I alleles may have an increased risk of carcinogenesis, especially in tumors associated with viral infections, such as HPV‐related cervical cancer (Reeves et al. 2019; Wu and Zhao 2021).

In this study, we propose that the presence of specific *ERAP1* variants and haplotypes may be associated with reduced survival in patients with cervical cancer. The aim of this study was to investigate the correlation between these genetic variants and haplotypes and the clinical prognosis of cervical cancer patients across populations using two cohorts of cervical cancer patients, thus providing a comprehensive understanding of the impact of these variants on survival.

## 2 Materials and Methods

### 2.1 Study Design and Data Collection

This study evaluated the association between *ERAP1* (*OMIM: 606832; NCBI Reference Sequence: NM_016442.5*) variants and haplotypes and the prognosis of cervical cancer using two independent datasets: (1) whole‐exome sequencing (WES) data from 173 cervical cancer patients, and relevant clinical data (Table S1) retrieved from the TCGA database for cervical squamous cell carcinoma and endocervical adenocarcinoma (CESC), available through the Genomic Data Commons (GDC) portal (accession code: phs000178.v9.p8); and (2) Sanger sequencing of the *ERAP1* gene in 52 tumor samples from Brazilian cervical cancer patients, and relevant clinical data (Table S2) enrolled in a retrospective cohort via convenience sampling. Patients were recruited at Presidente Dutra University Hospital (Federal University of Maranhão, HU/UFMA) between August 2022 and March 2023. Only patients who did not undergo any treatment before sample collection were included in the study. This study was approved by the Ethical Review Board of the René Rachou Institute/Fiocruz (approval number 5.534.968), and informed consent was obtained from all study subjects.

### 2.2 Identification of *ERAP1* Variants and Haplotypes

#### 2.2.1 TCGA‐CESC Cohort

The collection and DNA extraction of primary tumor tissue and blood samples, capture and sequencing of exons, quality control, and alignment of the sequences to the GRCh38.d1.vd1 version of the reference human genome have been described by The Cancer Genome Atlas Research (TCGA‐CESC) Network (The Cancer Genome Atlas Research Network 2017). *ERAP1* haplotypes were determined from WES data of both tumor and normal (blood) samples from the TCGA‐CESC cohort. *SAMtools Mpileup* (Li et al. 2009) version 1.3.1 was used to identify consistent mismatches at a single base pair resolution between the mapped reads on the reference genome at the *ERAP1* gene *locus* (chr5: 96,760,810–96,808,100), with minimum established coverage (−Q 25). *BCFtools call* was used to perform multiallelic and rare variant calling to identify *ERAP1* variants. To determine the haplotypes, *Beagle* software version 5.0 was used for genotype phasing and imputation of potential non‐genotyped markers, using the 1000 Genomes Project phase 3 reference panel (Devuyst 2015) and genetic maps from HapMap, both aligned to the GRCh38 version of the human genome.

Considering the phased *ERAP1* genotype, *BCFtools csq* (Danecek et al. 2021) version 1.9‐129‐g81ce96d was used for haplotype‐aware consequence calling of the detected haplotypes. The annotation file in compressed GFF3 format referring to the GRCh38 version of the human genome was obtained from the *Ensembl* database. Outcomes were selected based on the two most common transcripts of *ERAP1*: ENST00000296754 and ENST00000443439. Only missense variants located in coding exons were assessed and used for haplotype assembly. Table 1 summarizes the *ERAP1* variants that were selected for subsequent analysis. Given the high polymorphic nature of *ERAP1*, we standardized the haplotype nomenclature to allow for the identification of unique combinations of variants in the same allele. We established the nomenclature “H000”, where the three digits identify the different haplotypes. The wild‐type (WT) *ERAP1* haplotype was classified as H001.

**TABLE 1:** Summary of the *ERAP1* variants identified in this study, including their genomic positions, altered alleles, and amino acid changes (according to the GRCh38 human reference genome).

Exon	dbSNP	Localization	Alleles	HGVSc	HGVSp
2	rs72773968	96803892	G>A	c.35C>T	p.Thr12Ile
2	rs3734016	96803761	G>A	c.166G>A	p.Glu56Lys
2	rs26653	96803547	C>A,G,T	c.380G>C	p.Arg127Pro
5	rs26618	96795133	T>A,C,G	c.828A>G	p.Ile276Met
6	rs27895	96793840	C>A,G,T	c.1037G>A	p.Gly346Asp
6	rs2287987	96793832	T>A,C	c.1045A>G	p.Met349Val
11	rs30187	96788627	T>A,C	c.1583A>G	p.Lys528Arg
12	rs10050860	96786506	C>T	c.1723G>A	p.Asp575Asn
15	rs17482078	96783162	C>G,T	c.2174G>A	p.Arg725Gln
15	rs27044	96783148	G>A,C	c.2188C>G	p.Gln730Glu

#### 2.2.2 Brazilian Cervical Cancer Cohort

Tumor samples from cervical cancer patients were subsequently stored in RNALater and preserved at −80°C until processing. Genomic DNA was extracted using Wizard Genomic DNA Purification (Promega), according to the manufacturer's instructions. Six *ERAP1* variants, namely rs72773968, rs2287987, rs30187, rs10050860, rs17482078, and rs27044, were selected for evaluation. The genetic variants analyzed in the Brazilian dataset were selected based on their presence in haplotype H005, which was associated with worse survival in the *in silico* analysis of the TCGA cervical cancer dataset. These variants were chosen based on a review of previous publications and search results from the *NCBI Database of Short Genetic Variations (dbSNP)*, highlighting their association with reduced survival and lymph node metastasis in cervical cancer patients (Mehta et al. 2009, 2007; Reeves et al. 2013, 2019).

Specific primers were designed to amplify the *ERAP1* exon regions containing the variants, using Primer3plus software. The OligoAnalyzer online tool (*idtdna.com/calc/analyzer*) was used to verify parameters, such as secondary structure formation, primer dimer formation, G‐C content percentage, and annealing temperature. The specificity of primer binding to the regions of interest was evaluated by local alignment using BLAST and Smith–Waterman software. The *ERAP1* sequence used for primer design was obtained from the reference human genome GRCh38, which was the same sequence used in the *in silico* steps to identify the target variants. Five primer pairs were used to amplify the regions of interest in the *ERAP1* gene.

High‐fidelity Platinum Taq *DNA* Polymerase was used to amplify the regions of interest (Table S4). The quality of the amplification was evaluated by electrophoresis on a 1% agarose gel, with an expected mean amplicon length of 200bp. Subsequently, PCR amplicons of the correct size were purified using the QIAquick PCR Purification Kit (Qiagen). Sequencing of the *ERAP1* regions was performed using the BigDye Terminator v3.1 Cycle Sequencing Kit (Applied Biosystems), with the same primers as described previously, in an ABI 3730xL DNA analyzer (Thermo Fisher). Each sample was sequenced using both forward and reverse primers, with two sequences corresponding to the forward primer and two sequences corresponding to the reverse primer. BLAST analysis was performed to determine whether the generated consensus sequences corresponded to the target regions.

Sequence analysis was performed using Geneious Prime 2023.0.4 software. The presence of the variants was determined according to the following parameters: minimum coverage > 3, minimum variant frequency 0.25, maximum variant *p* value 10^−6^, and minimum strand‐bias *p* value 10^−5^, when exceeding 65% bias. The identification of heterozygosity was based on the presence of two peaks at the position of the variant of interest, with 80% peak similarity and 10% peak detection height. The genotype of each variant was confirmed by manual observation of fluorescence peaks (Figure 1).

**FIGURE 1:**
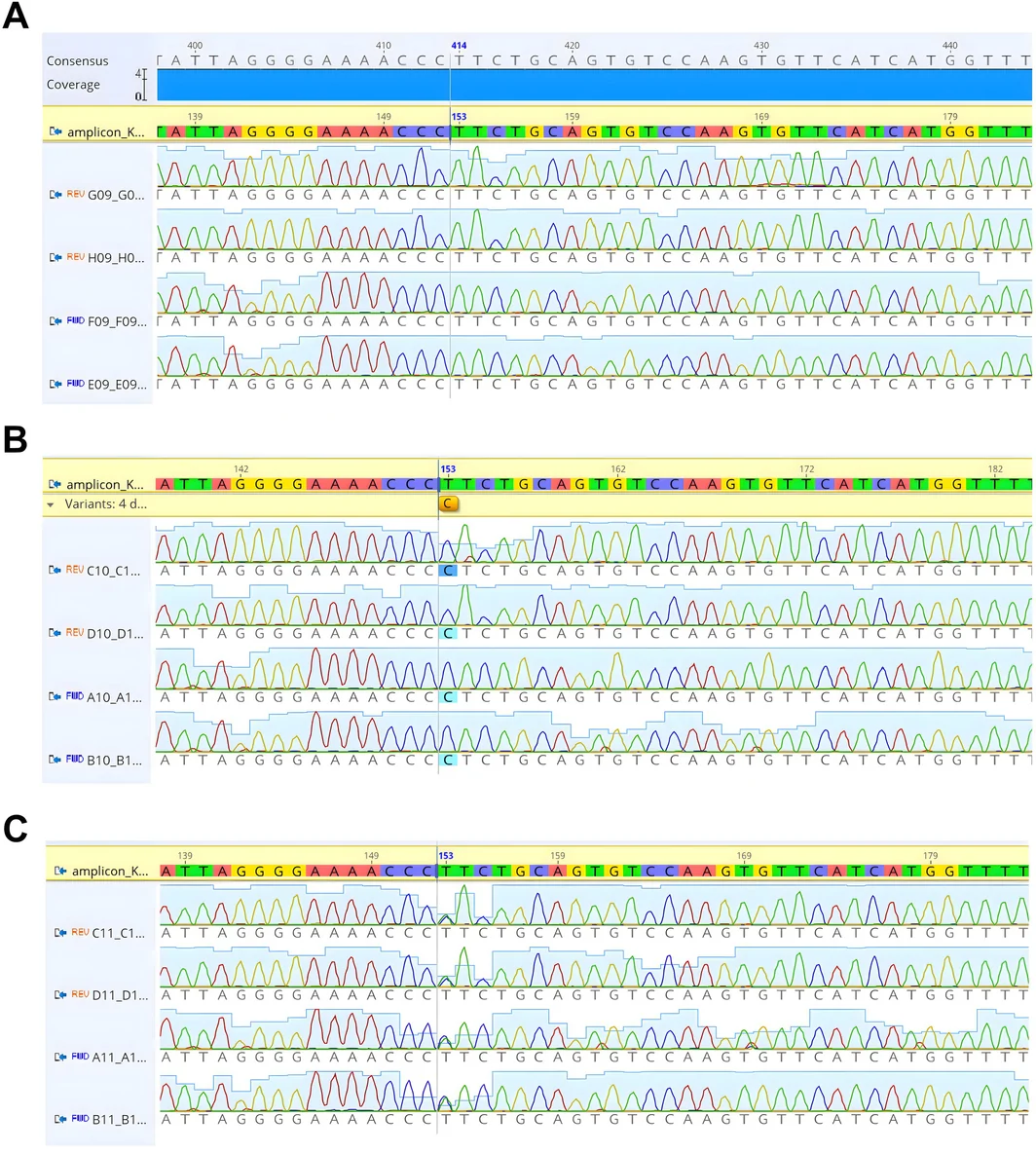
Example of chromatograms obtained by Sanger sequencing of *ERAP1* regions and identification of variants using *Geneious Prime* software. The example represents the identification of the rs30187 (p.Lys528Arg) variant, which is located at position 153 within the amplified DNA fragment. (A) Absence of rs30187 on all strands, (B) rs30187 present in the homozygous state, and (C) rs30187 present on at least one strand, indicating a heterozygous state.

#### 2.2.3 *HLA‐I* Typing in the TCGA‐CESC Dataset

*HLA* typing was further performed using WES data from both tumor and normal (blood) samples from TCGA‐CESC patients. Paired‐end DNA sequencing reads were separated into reads flagged as READ1 and READ2 using the *fastq* tool in the *SAMtools* package. Read mapping against the nucleotide coding sequence (CDS) reference library and filtering of sequencing data for *HLA‐I* reads were performed using *RazerS3* version 2.4.0. Only the best alignment for every read with a sequence identity of at least 95% was considered and used for downstream analysis. *HLA typing* was further performed using the *OptiType* algorithm (Szolek et al. 2014) version 1.3.1. Association analysis between *HLA‐I* and FIGO stages was performed within the TCGA dataset using the *PyHLA* tool (Fan and Song 2017) version 1.1.1. The χ^2^ test was used to assess the association between *HLA‐I* alleles and the FIGO stage, and the association was evaluated by calculating the odds ratio (OR). For all analyses, a confidence interval (CI) of 95% was established. Differences were considered statistically significant at *p* < 0.05.

### 2.3 Statistical Analysis

The association of overall survival (OS) and disease‐specific survival (DSS) with clinical characteristics and the presence of *ERAP1* variants and haplotypes was evaluated across the TCGA and the Brazilian cohorts. Clinical characteristics were stratified based on tumor stage (I, II, or III/IV) and age at diagnosis (< 49 or ≥ 49 years). OS was calculated as the time from diagnosis to the date of death from any cause or the last follow‐up, whereas disease‐specific survival (DSS) was defined as the time from diagnosis to the date of death specifically attributed to cervical cancer or the last follow‐up.

With respect to the TCGA cohort, analyses were conducted at both haplotype and variant levels, and only haplotypes with sufficient variability were included in the model, ensuring reliable estimates. The following variants were analyzed: rs3734016 (p.Glu56Lys), rs26653 (p.Arg127Pro), rs26618 (p.Ile276Met), rs27895 (p.Gly346Asp), rs72773968 (p.Thr12Ile), rs2287987 (p.Met349Val), rs30187 (p.Lys528Arg), rs10050860 (p.Asp575Asn), s17482078 (p.Arg725Gln), and rs27044 (p.Gln730Glu). In contrast, for the Brazilian cohort, analyses were performed only at the variant level, focusing on rs72773968 (p.Thr12Ile), rs2287987 (p.Met349Val), rs30187 (p.Lys528Arg), rs10050860 (p.Asp575Asn), s17482078 (p.Arg725Gln), and rs27044 (p.Gln730Glu) variants. Kaplan–Meier survival curves and log‐rank tests were used to evaluate the impact of each clinical and genetic variable on DSS and OS. Additionally, multivariate Cox proportional hazards regression analyses were employed to estimate the relative risk (hazard ratio, HR) and 95% confidence intervals (CI) for the associations of *ERAP1* variants, haplotypes, and clinical characteristics with clinical outcomes (DSS or OS). All survival analyses were performed using the survival package (version 2.44‐1.1), Survminer (version 0.4.5), and ggplot2 (version 3.2.0) in R (version 3.4.4).

### 2.4 Assessment of *ERAP1* Expression and Immune Infiltration

We used the Tumor IMmune Estimation Resource (TIMER) (Li et al. 2020) tool to evaluate the association of *ERAP1* expression with infiltrating immune cells, including CD4+ and CD8+ T cells, neutrophils, dendritic cells (DCs), B cells, and macrophages, in patients with cervical cancer from the TCGA dataset. A multivariate Cox proportional hazards analysis adjusted for age and FIGO stage was performed to explore the relationship between tumor immune cells and survival. Kaplan–Meier plots for immune infiltrates and *ERAP1* expression levels were generated to visualize survival differences.

## 3 Results

### 3.1 Determination of *ERAP1* Genetic Variants Among the Cervical Cancer Cohorts

In the TCGA‐CESC cohort, *in silico* analysis identified missense germline variants in the *ERAP1* gene. Nearly all allele frequencies (AFs) from the cervical cancer patient data were positively correlated with those from the reference panel (Figure S1A,B). The frequency of variants within the exome of blood and tumor samples exhibited similar frequency profiles (Figure S1C). The analyzed samples covered at least 32 Mb of exonic regions, with an average coverage of 49× (range: 7–351×) for tumor samples and 47× (range: 9–341×) for blood samples (The Cancer Genome Atlas Research Network 2017). All *ERAP1* variants demonstrated a median coverage greater than 34× (Table S3).

Among the TCGA cohort, the rs30187 (p.Lys528Arg) variant exhibited the highest minor allele frequency (MAF) (39.3%), followed by rs26653 (p.Arg127Pro) (32.4%), rs27044 (p.Gln730Glu) (29.2%), rs26618 (p.Ile276Met) (26.3%), rs10050860 (p.Asp575Asn) and rs17482078 (p.Arg725Gln) (both with 15.9%), and rs2287987 (p.Met349Val) (14.5%). Variants with lower MAFs included rs72773968 (p.Thr12Ile) (9.5%), rs3734016 (p.Glu56Lys) (6.4%), and rs27895 (p.Gly346Asp) (3.5%).

To investigate *ERAP1* variants in Brazilian cervical cancer patients, we selected six variants located within key haplotypes previously associated with poor prognosis in the haplotype‐level analysis of the TCGA‐CESC cohort. Moreover, these variants constitute the haplotype identified by Reeves et al. (2013) as encoding a nonfunctional *ERAP1* protein, which impairs the generation of specific antigenic epitopes and thereby contributes to immune evasion. This selection was also based on a review of previous publications and search results from the *NCBI Database of Short Genetic Variations* (dbSNP), highlighting their association with reduced survival and lymph node metastasis in cervical cancer patients (Mehta et al. 2007, 2009; Reeves et al. 2013, 2019). As a result, six variants were identified for analysis, including: rs72773968, rs2287987, rs30187, rs10050860, rs17482078, and rs27044.

In the cohort of Brazilian patients, rs30187 (p.Lys528Arg) variant had the highest MAF (49%), followed by rs27044 (p.Gln730Glu) (30.8%), and rs10050860 (p.Asp575Asn) (16.3%). Variants with lower MAFs included rs17482078 (p.Arg725Gln) (14.4%), rs2287987 (p.Met349Val) (13.5%), and rs72773968 (p.Thr12Ile) (9.6%).

We further compared the MAFs of these variants between the two cohorts and the allele frequencies reported in the AbraOM database (Naslavsky et al. 2022), a repository of genomic variants derived from a cohort of elderly Brazilian individuals (Table 2). Among the six variants evaluated in both cohorts, most showed consistent MAFs across datasets, including rs72773968 (p.Thr12Ile), rs10050860 (p.Asp575Asn), rs17482078 (p.Arg725Gln), and rs2287987 (p.Met349Val). However, significant variability was observed for rs30187 (p.Lys528Arg), which had a MAF of 39.3% in the TCGA, 49% in the Brazilian cohort, and 39.6% in the AbraOM database. The four variants evaluated only in the TCGA cohort, when compared to the AbraOM database, displayed distinct frequency differences. For instance, rs3734016 (p.Glu56Lys), rs26653 (p.Arg127Pro), rs26618 (p.Ile276Met), and rs27895 (p.Gly346Asp) showed lower MAFs in the TCGA cohort (6.4%, 32.4%, 26.3%, and 3.5%, respectively), compared to their respective frequencies in the AbraOM database (40.99%, 64.43%, 22.33%, and 70.88%, respectively).

**TABLE 2:** Distribution of *ERAP1* variants identified in the TCGA and a Brazilian cohort of cervical cancer, including the minor allele frequencies (MAFs) identified in this study and the MAFs retrieved from the AbraOM database.

TCGA cohort						Brazilian cohort						
Variant		Genotype	N (%)	N total (%)	MAFs (MA)	Variant		Genotype	N (%)	N total (%)	MAFs (MA)	MAF AbraOM
rs72773968	p.Thr12Ile	G/G	141 (81.5)	173 (100)	9,5% (A)	rs72773968	p.Thr12Ile	G/G	47 (90.3)	52 (100)	9.6% (A)	12.34%
		G/A	31 (17.9)					G/A	5 (9.6)			
		A/A	1 (0.6)					—	—			
rs2287987	p.Met349Val	T/T	126 (72.8)	173 (100)	14.5% (C)	rs2287987	p.Met349Val	T/T	40 (76.9)	52 (100)	13.5% (C)	16.86¨%
		T/C	44 (25.4)					T/C	10 (19.2)			
		C/C	3 (1.7)					C/C	2 (3.8)			
rs30187	p.Lys528Arg	T/C	84 (48.6)	173 (100)	39.3% (T)	rs30187	p.Lys528Arg	C/C	15 (28.8)	52 (100)	49.0% (T)	59.05%
		C/C	63 (36.4)					C/T	23 (44.2)			
		T/T	26 (15.0)					T/T	14 (26.9)			
rs10050860	p.Asp575Asn	C/C	123 (71.1)	173 (100)	15.9% (T)	rs10050860	p.Asp575Asn	C/C	38 (73.08)	52 (100)	16.3% (T)	16.95%
		C/T	45 (26.0)					C/T	11 (21.1)			
		T/T	5 (2.9)					T/T	3 (5.7)			
rs17482078	p.Arg725Gln	C/C	123 (71.1)	173 (100)	15.9% (T)	rs17482078	p.Arg725Gln	C/C	40 (76.9)	52 (100)	14.4% (T)	16.22%
		C/T	45 (26.0)					C/T	9 (17.3)			
		T/T	5 (2.9)					T/T	3 (5.7)			
rs27044	p.Gln730Glu	C/C	85 (49.1)	173 (100)	29.2% (G)	rs27044	p.Gln730Glu	C/C	25 (48.08)	52 (100)	30.8% (G)	67.84%
		G/C	75 (43.4)					G/C	22 (42.31)			
		G/G	13 (7.5)					G/G	5 (9.62)			
rs3734016	p.Glu56Lys	C/C	151 (87.3)	173 (100)	6.4% (T)	—						40.99%
		C/T	22 (12.7)									
rs26653	p.Arg127Pro	G/G	79 (45.7)	173 (100)	32.4% (C)	—						64.43%
		C/G	76 (43.9)									
		C/C	18 (10.4)									
rs26618	p.Ile276Met	T/T	94 (54.3)	173 (100)	26.3% (C)	—						22.33%
		T/C	67 (38.7)									
		C/C	12 (6.9)									
rs27895	p.Gly346Asp	C/C	161 (93.1)	173 (100)	3.5% (T)	—						70.88%
		C/T	12 (6.9)									

*Note:* Six variants were evaluated in both cohorts, while four were assessed only in the TCGA cohort. MAFs from the AbraOM database, representing healthy Brazilian individuals, are included for comparison.

### 3.2 In Silico Determination of *ERAP1* Haplotypes Among TCGA‐CESC Patients

Haplotype assembly was performed using the tumor WES data from the TCGA‐CESC cohort, focusing on variants present in more than 1% of patients. These included the following variants: rs72773968, rs3734016, rs26653, rs26618, rs27895, rs2287987, rs30187, rs10050860, rs17482078, and rs27044 (Figure S1C). The analysis identified 52 different haplotypes in 81 unique combinations (Table S5). H018 was the most frequently detected haplotype, with 55 occurrences (Figure S1D). This haplotype is composed of rs26653, rs30187, and rs27044 variants, which are the most frequently detected *ERAP1* variants in patients with cervical cancer. H001, corresponding to the WT haplotype, was the second most frequently detected haplotype, with 34 occurrences. The most common combination of haplotypes was homozygosity for H018 (H018‐H018), followed by H001‐H018 and H001‐H001 combinations (Figure S1E). The haplotypes of each patient, as well as the genetic variants that compose them, are described in Table S6.

### 3.3 Survival Analysis

#### 3.3.1 The Impact of *ERAP1* Variants on the Clinical Outcome of TCGA and Brazilian Cervical Cancer Patients

In the TCGA cohort, rs2287987 (p.Met349Val) was significantly associated with OS (*p* = 0.028) and DSS (*p* = 0.009) (Figure 2A,B). The rs30187 (p.Lys528Arg) variant showed a borderline association with OS (*p* = 0.056)(Figure 2D), but no significant association with DSS (*p* = 0.130). Other variants, including rs3734016 (p.Glu56Lys), rs26653 (p.Arg127Pro), and rs72773968 (p.Thr12Ile), were not significantly associated with OS or DSS (*p* > 0.05). Regarding the clinicopathological characteristics, advanced FIGO stage (III/IV) was significantly associated with OS (*p* = 0.043), indicating a strong impact on the overall survival of TCGA patients (Figure 2C). With respect to the cohort of Brazilian patients, none of the *ERAP1* variants analyzed were significantly associated with OS or DSS (*p* > 0.05). The complete results of the Kaplan–Meier analysis with the log‐rank test for TCGA and Brazilian cervical cancer patients are presented in Tables S7 and S8.

**FIGURE 2:**
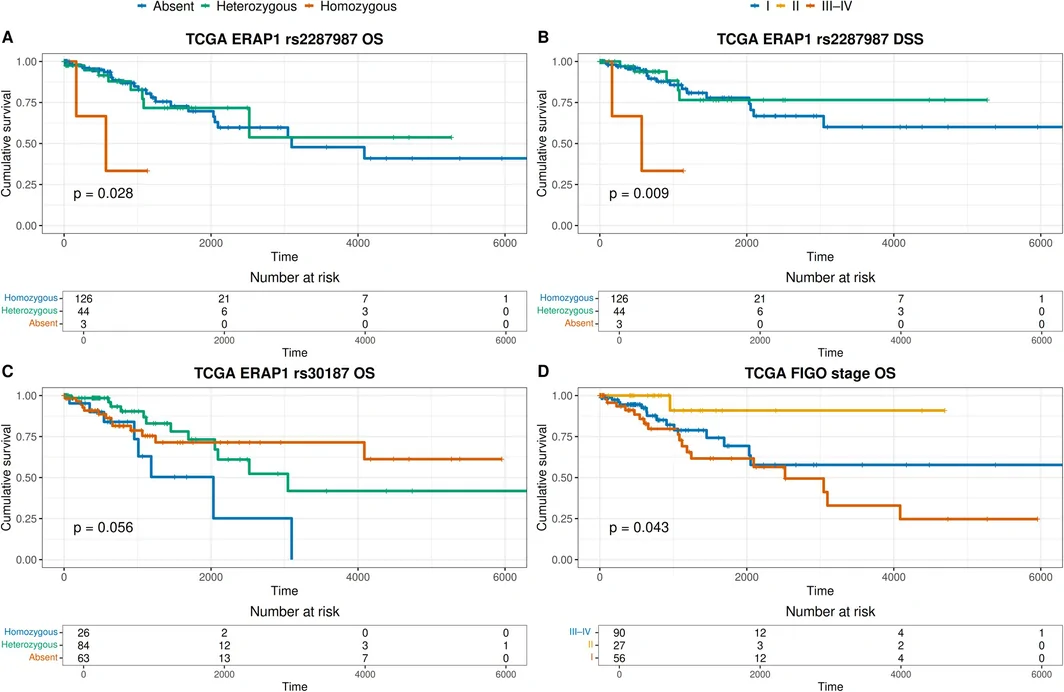
Survival curves for *ERAP1* rs2287987 (p.Met349Val) variant and FIGO staging in TCGA cervical cancer patients. (A) Kaplan–Meier curve for overall survival (OS) stratified by the presence of *ERAP1* rs2287987 variant. Patients are categorized into three groups: Absent (yellow line), heterozygous (blue line), and homozygous (red line). The log‐rank test indicated a significant association between the variant and OS (*p* = 0.028). (B) Kaplan–Meier curve for disease‐specific survival (DSS) for the same variant. Similar stratification revealed a significant association between the variant and DSS (*p* = 0.009). The number of patients at risk at different times is indicated at the bottom of each plot. (C) Kaplan–Meier curve for overall survival (OS) stratified by FIGO stage (I, II, and III/IV). (D) Kaplan–Meier curve for overall survival (OS) stratified by FIGO stage (I, II, and III/IV). Patients in FIGO stages I (yellow line), II (blue line), and III/IV (red line) show significant differences in survival, with more advanced stages associated with a decreased survival probability. The log‐rank test indicated a significant association between FIGO stage and OS (*p* = 0.043).

Multivariate Cox regression analysis was conducted to evaluate the associations of *ERAP1* variants and clinicopathological variables with OS and DSS in both TCGA and Brazilian cervical cancer patients. In the TCGA cohort, the homozygous state of the rs2287987 (p.Met349Val) variant was associated with a significantly increased risk of death in both OS (HR = 11.0434, 95% CI = 2.1757–56.0533, *p* = 0.00376) (Table 3) and DSS (HR = 13.833, 95% CI = 2.5112–76.199, *p* = 0.00255) (Table 4). In terms of OS, rs30187 (p.Lys528Arg) variant was significantly associated with a reduced risk of mortality in both the heterozygous (HR = 0.3740, 95% CI = 0.1460–0.9586, *p* = 0.04055) and homozygous (HR = 0.3463, 95% CI = 0.1269–0.9452, *p* = 0.03845) forms. In the cohort of Brazilian cervical cancer patients, no statistically significant associations between *ERAP1* variants and OS or DSS were observed (*p* > 0.05) (Tables S9 and S10).

**TABLE 3:** Cox regression analysis for disease‐specific survival (DSS) in the TCGA cohort of cervical cancer patients considering *ERAP1* variants and clinicopathological variables.

Variable			Overall survival					
			Univariate			Multivariate		
			HR	95% CI	*p*	HR	95% CI	*p*
rs3734016	p.Glu56Lys	Heterozygous	5.742	0.1747–1.887	0.361			
rs26653	p.Arg127Pro	Heterozygous	7.381	0.2440–2.233	0.591			
		Homozygous	4.237	0.1352–1328	0.141			
rs72773968	p.Thr12Ile	Heterozygous	0.9205	0.3789–2.237	0.855			
		Homozygous	0.0000008092	0–Inf	0.997			
rs26618	p.Ile276Met	Heterozygous	81.433	0.3894–1.703	0.585			
		Homozygous	1.04506	0.2433–4.488	0.953			
rs27895	p.Gly346Asp	Heterozygous	1.8801	0.5672–6.232	0.302			
rs2287987	p.Met349Val	Heterozygous	9.451	0.4223–2.115	0.8906			
		Homozygous	5.6791	1.3040–24.734	0.0207*	11.0434	2.1757–56.0533	0.00376*
rs30187	p.Lys528Arg	Heterozygous	0.3723	0.1510–0.9177	0.0318*	0.3740	0.1460–0.9586	0.04055*
		Homozygous	0.3981	0.1619–0.9787	0.0448*	0.346	0.1269–0.9452	0.03845*
rs10050860	p.Asp575Asn	Heterozygous	0.9799	0.4375–2.195	0.961			
		Homozygous	1.3540	0.3178–5.769	0.682			
rs17482078	p.Arg725Gln	Heterozygous	0.8675	0.3726–2.020	0.742			
		Homozygous	1.3169	0.3098–5.597	0.709			
rs27044	p.Gln730Glu	Heterozygous	0.5933	0.1928–1.826	0.363			
		Homozygous	0.4844	0.1609–1.459	0.197			
FIGO stage	I v. III_IV		2.0147	1.011–4.014	0.0464*	2.1186	1.0276–4.3678	0.04198*
Age	< 49 y vs. > 49 y		1.355	0.6807–2.696	0.387			

*Note:* The table presents the hazard ratios (HRs), 95% confidence intervals (CIs), and *p*‐values for each variant and variable in both the initial and final models. Statistically significant results are marked with an asterisk (*), indicating a *p*‐value < 0.05.

**TABLE 4:** Cox regression analysis for disease‐specific survival (DSS) in the TCGA cohort of cervical cancer patients considering *ERAP1* variants and clinicopathological variables.

Variable			Disease‐specific survival					
			Univariate			Multivariate		
			HR	95% CI	*p*	HR	95% CI	*p*
rs3734016	p.Glu56Lys	Heterozygous	0.2479	0.03342–1.84	0.173			
rs26653	p.Arg127Pro	Heterozygous	0.8592	0.2427–3.042	0.814			
		Homozygous	0.3865	0.1010–1.479	0.165			
rs72773968	p.Thr12Ile	Heterozygous	1.091	0.4062–2.932	0.862			
		Homozygous	0.0000008342	0–Inf	0.998			
rs26618	p.Ile276Met	Heterozygous	1.049	0.4648–2.368	0.908			
		Homozygous	0.00000003856	0—Inf	0.997			
rs27895	p.Gly346Asp	Heterozygous	2.529	0.749–8.542	0.135			
rs2287987	p.Met349Val	Heterozygous	0.7969	0,2937–2162	0,6558			
		Homozygous	6.6808	1.5042–29,673	0.0125*	13.833	2.5112–76.199	0.00255*
rs30187	p.Lys528Arg	Heterozygous	0.3905	0.1381–1.105	0.763			
		Homozygous	0.4044	0.1422–1.150	0.895	0.3483	0.1076–1.128	0.07863
rs10050860	p.Asp575Asn	Heterozygous	0.8068	0.2972–2.190	0.674			
		Homozygous	1.9226	0.4429–8.346	0.383			
rs17482078	p.Arg725Gln	Heterozygous	0.8462	0.3114–2.300	0.743			
		Homozygous	1.9468	0.4485–8.451	0.374			
rs27044	p.Gln730Glu	Heterozygous	0.6097	0.1673–2.222	0.453			
		Homozygous	0.4677	0.1301–1.681	0.244			
FIGO stage	I vs. III_IV		2.018	0.8993–4.526	0.0887	0.3484	0.1076–1.128	0.06404
Age	< 49 y vs. > 49 y		1.107	0.496–2.469	805			

*Note:* The table presents the hazard ratios (HRs), 95% confidence intervals (CIs), and *p*‐values for each variant and variable in both the initial and final models. Statistically significant results are marked with an asterisk (*), indicating a *p*‐value < 0.05.

#### 3.3.2 Impact of *ERAP1* Haplotypes on the Clinical Outcome of TCGA Cervical Cancer Patients

In line with the results of the variant‐level analysis, Kaplan–Meier survival analysis with the log‐rank test was performed to assess the association of *ERAP1* haplotypes with OS and DSS in cervical cancer patients from TCGA. Notably, haplotype H023 was significantly associated with both OS (*p* = 0.029) and DSS (*p* = 0.0085), indicating that patients carrying this haplotype may have a poorer prognosis (Figure 3C,D). The presence of haplotype H005 was not associated with survival outcomes; however, its presence in the homozygous state was significantly associated with both OS (*p* = 0.027) and DSS (*p* = 0.0088) (Figure 3A,B).

**FIGURE 3:**
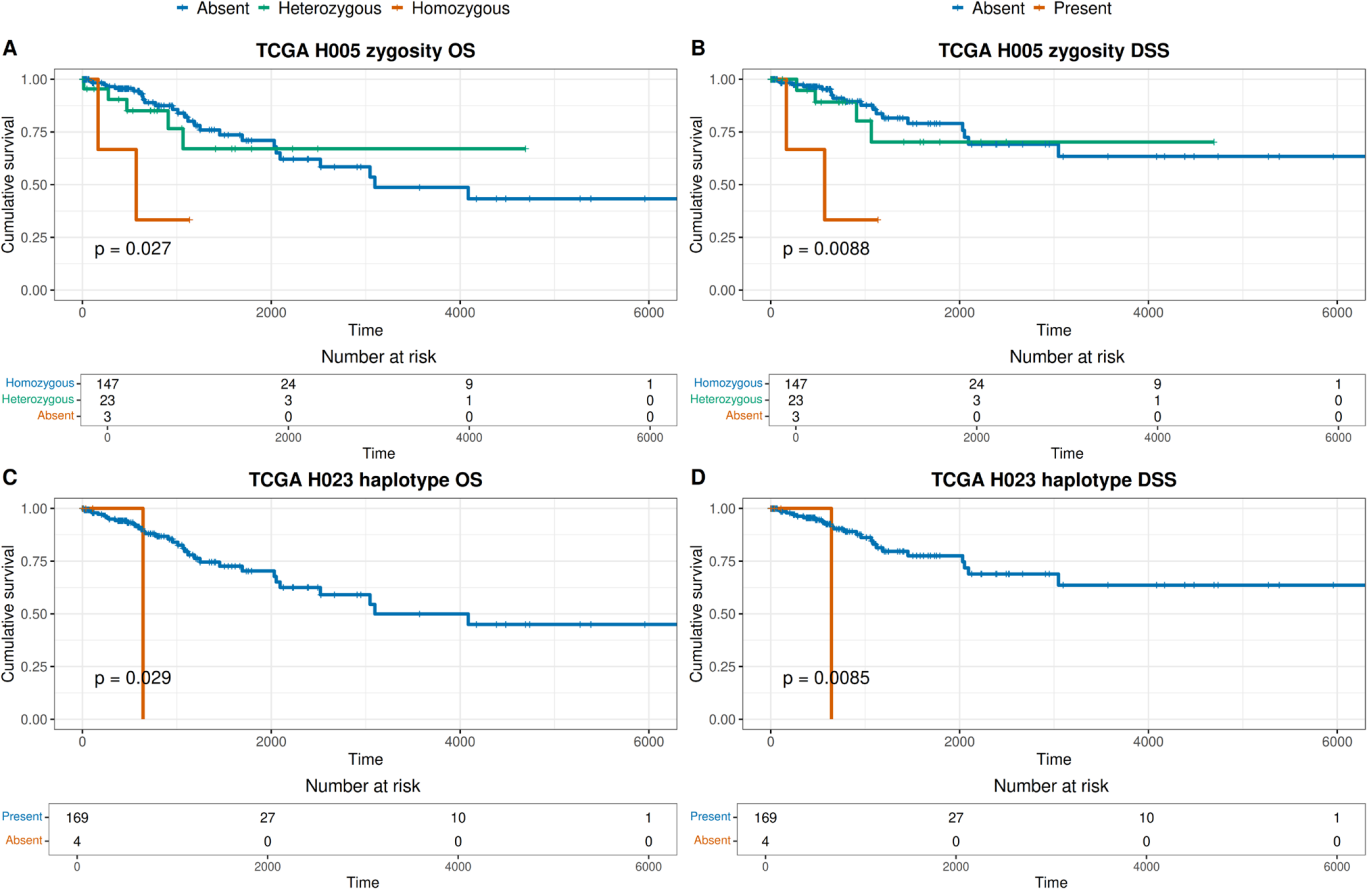
Survival curves for overall survival (OS) and disease‐specific survival (DSS) based on the presence of H023 and the homozygosity of *ERAP1* H005 haplotype in TCGA‐CESC cervical cancer patients. (A) Kaplan–Meier curve for overall survival (OS) stratified by the same H005 haplotype groups. H005 homozygosity was also significantly associated with OS (p = 0.027). (B) Kaplan–Meier curve for disease‐specific survival (DSS) stratified by the presence of H005 haplotype. Patients are categorized into three groups: Absent (yellow line), heterozygous (blue line), and homozygous (red line). H005 homozygosity was significantly associated with DSS (p = 0.0088). (C) Kaplan–Meier curve for overall survival (OS) stratified by the same groups, showing a significant association between H023 haplotype and OS (p = 0.029). (D) Kaplan–Meier curve for disease‐specific survival (DSS) stratified by the presence (blue line) or absence (yellow line) of H023 haplotype. The log‐rank test indicated a significant association between the presence of H023 and DSS (p = 0.0085).

Multivariate Cox regression analysis revealed an association between H005 and H023 haplotypes and both OS (Table 5) and DSS (Table 6). H005 homozygosity was associated with a greater risk of both overall (HR = 9.591, 95% CI = 2.0713–44.412, *p* = 0.00384) and disease‐specific death (HR = 12.8510, 95% CI = 2.6277–62.850, *p* = 0.00162). Additionally, H023 haplotype was significantly associated with worse survival outcomes, with an increased risk of both OS (HR = 10.183, 95% CI = 1.2744–81.374, *p* = 0.02862) and DSS (HR = 15.183, 95% CI = 1.8223–126.493, *p* = 0.01191). Furthermore, FIGO stage III/IV disease was a significant predictor of poorer outcomes, with an increased risk of both OS (HR = 2.412, 95% CI = 1.1658–4.991, *p* = 0.01763) and DSS (HR = 2.628, 95% CI = 1.1001–6.277, *p* = 0.02965) (Table 5, 6).

**TABLE 5:** Multivariate Cox regression analysis for overall survival (OS) in TCGA‐CESC cohort of cervical cancer patients considering *ERAP1* haplotypes and clinicopathological variables.

Variable		Overall survival					
		Univariate			Multivariate		
		HR	95% CI	*p*	HR	95% CI	*p*
H001—*wt*		1.916	0.8523–4.305	0.116			
H002: rs72773968—rs26653		1.8913	0.3805–3.118	0.874			
H004: rs26653—rs2287987—rs30187—rs10050860		1.6816	0.227–12.46	0.611			
H005: rs26653—rs2287987—rs30187—rs10050860—rs17482078—rs27044		1.5348	0.663–3.553	0.317			
H005: rs26653—rs2287987—rs30187—rs10050860—rs17482078—rs27044	Heterozygous	1.1893	0.455–3.108	0.7236			
	Homozygous	5.9323	1.370–25.689	0.0173*	9.591	2.0713–44.412	0.00384*
H006: rs26618—rs30187—rs27044		1.906	0.5729–64.311	0.291			
H007: rs26653		0.6622	0.08989–4.877	0.686			
H010: rs26653—rs30187—rs27044		0.8714	0.3581–2.12	0.762			
H013: rs26653—rs2287987—rs30187—rs10050860—rs27044		0.6698	0.0911–4.925	0.694			
H014: rs27044		0.8631	0.3323–2.242	0.762			
H017: rs27895—rs30187		1.9822	0.2679–14.67	0.503			
H018: rs26653—rs26618—rs30187—rs27044		1.099	0.5099–2.369	0.81			
H019: rs3734016—rs26653—rs30187—rs27044		0.5262	0.1257–2.203	0.38			
H020: rs27895—rs30187—rs27044		2.1321	0.288–15.78	0.459			
H021: rs72773968—rs26653—rs27044		1.6968	0.4023–7.157	0.472			
H022: rs17482078—rs27044		4255	0.5683–31.87	0.159			
H023: rs26653—rs27895—rs30187—rs27044		7039	0.9155–54.12	0.0608*	10.183	1.2744–81.374	0.02862*
H038: rs26653—rs27044		14,744	0.198–10.98	0.705			
H046: rs30187		27,068	0.2167–11.99	0.33			
FIGO stage	I vs. III_IV	2.0147	1.011–4.014	0.0464*	2.412	1.1658–4.991	0.01763*
Age	< 49 y vs. > 49 y	1.3546	0.6807–2.696	0.387			

*Note:* The table presents the hazard ratios (HR), 95% confidence intervals (CIs), and *p*‐values for each haplotype and variable in both the initial and final models. Statistically significant results are marked with an asterisk (*), indicating a *p*‐value < 0.05.

**TABLE 6:** Multivariate Cox regression analysis for disease‐specific survival (DSS) in TCGA‐CESC cohort of cervical cancer patients considering *ERAP1* haplotypes and clinicopathological variables.

Variable		Disease‐specific survival					
		Univariate			Multivariate		
		HR	95% CI	*p*	aHR	95% CI	*p*
H001—wt		1.4699	0.5434–3.976	0.448			
H002: rs72773968—rs26653		1.1222	0.3332–3.78	0.852			
H004: rs26653—rs2287987—rs30187—rs10050860		2.2425	0.2987–16.83	0.432			
H005: rs26653—rs2287987—rs30187—rs10050860—rs17482078—rs27044		1.8006	0.7122–4.552	0.214			
H005: rs26653—rs2287987—rs30187—rs10050860—rs17482078—rs27044	Heterozygous	1.3117	0.4428–3.866	0.62435			
	Homozygous	7.4139	1.6750–32.807	0.00829*	12.8510	2.6277–62.850	0.00162*
H006: rs26618—rs30187—rs27044		2.4575	0.7283–8.292	0.147			
H007: rs26653		0.8275	0.1112–6.156	0.853			
H010: rs26653—rs30187—rs27044		0.8075	0.2747–2.373	0.697			
H014: rs27044		0.6594	0.1964–2.214	0.5			
H017: rs27895—rs30187		2.5943	0.3464–19.43	0.353			
H018: rs26653—rs26618—rs30187—rs27044		1.2186	0.505–2.941	0.66			
H020: rs27895—rs30187—rs27044		2.917	0.3892–21.86	0.298			
H021: rs72773968—rs26653—rs27044		2.638	0.6169–11.28	0.191			
H022: rs17482078—rs27044		5.384	0.7094–40.86	0.104			
H023: rs26653—rs27895—rs30187—rs27044		9.555	1.217–75.01	0.0318*	15.183	1.8223–126.493	0.01191*
H038: rs26653—rs27044		1.9135	0.2537–14.43	0.529			
H045: rs3734016—rs26653—rs27044		2.0547	0.2733–15.45	0.484			
H046: rs30187		3.458	0.4612–25.94	0.227			
FIGO stage	I vs. III_IV	2.018	0.8993–4526	0.0887	2.628	1.1001–6.277	0.02965*
Age	< 49 y vs. > 49 y	1.1067	0.496–2.469	0.805			

*Note:* The table presents the hazard ratios (HR), 95% confidence intervals (CIs), and *p*‐values for each haplotype and variable in both the initial and final models. Statistically significant results are marked with an asterisk (*), indicating a *p*‐value < 0.05.

**TABLE 7:** Multivariate Cox regression analysis of immune cell infiltration and *ERAP1* expression in TCGA‐CESC cohort of cervical cancer patients.

Variable	Multivariate analysis		
	HR	95% CI	*p*
Age	1.013	0.996–1.031	0.137
B cell	0.063	0–654.673	0.558
CD8 + T cell	0.006	0–0.969	0.049*
CD4+ T cell	0.001	0–4.819	0.114
Macrophage	16.291	0.012–22115.360	0.448
Neutrophil	0.007	0–106.879	0.314
Dendritic	58.277	0.444–7641.168	0.102
*ERAP1* expression	1.312	0.969–1.777	0.079

### 3.4 Association of HLA‐I Alleles With Cervical Cancer Risk Among TCGA Patients

*HLA‐I* genotyping of TCGA cervical cancer patients was performed. Among the *HLA‐A* loci, HLA‐A*02:01 allele was the most frequently detected (33.95%), followed by HLA‐A*01:01 (25.43%) and HLA‐A*03:01 (20.81%) alleles (Figure S2A). Considering the *HLA‐C* loci, HLA‐C*07:02 allele was the most common (28.32%), followed by HLA‐C*07:01 (22.54%) and HLA‐C*04:01 (20.23%) alleles (Figure S2B). The *HLA‐C* locus presented greater allelic variability than did the *HLA‐A* and *HLA‐B* loci, with 64 alleles detected. HLA‐B*07:02 was the most frequent allele (23.12%), followed by HLA‐B*08:01 (17.34%), and HLA‐B*44:02 (14.45%) alleles (Figure S2C).

No statistically significant associations were identified between *HLA‐I* alleles and FIGO stage (Table S11), nor was there any pattern of *HLA‐I* alleles among patients with *ERAP1* haplotypes associated with survival in cervical cancer patients.

### 3.5 Correlation Between *ERAP1* Expression and Immune Infiltration

We evaluated the correlation between *ERAP1* expression levels (log_2_ TPM) and tumor purity and immune cell infiltration, including that of CD8+ T cells, CD4+ T cells, macrophages, neutrophils, B cells, and dendritic cells, in cervical cancer data from the TCGA‐CESC cohort using TIMER software (Figure 5). A significant inverse correlation was observed between *ERAP1* expression and tumor purity (correlation = −0.13, *p* = 3.03e‐02), indicating that *ERAP1* expression tended to be higher in samples with greater immune cell content. Positive correlations with CD8+ T cells (partial correlation = 0.264, *p* = 0.0000096), CD4+ T cells (partial correlation = 0.312, *p* = 0.000000111), neutrophils (partial correlation = 0.483, *p* = 00000001), and dendritic cells (partial correlation = 0.342, *p* = 0.0000000052) suggested that elevated *ERAP1* expression was associated with increased infiltration of these immune cell types. In contrast, the correlations between macrophages (partial correlation = −0.074, *p* = 0.217) and B cells (partial correlation = 0.092, *p* = 0.125) were not statistically significant, implying a weaker association between *ERAP1* expression and these immune cells within the tumor microenvironment.

**FIGURE 4:**
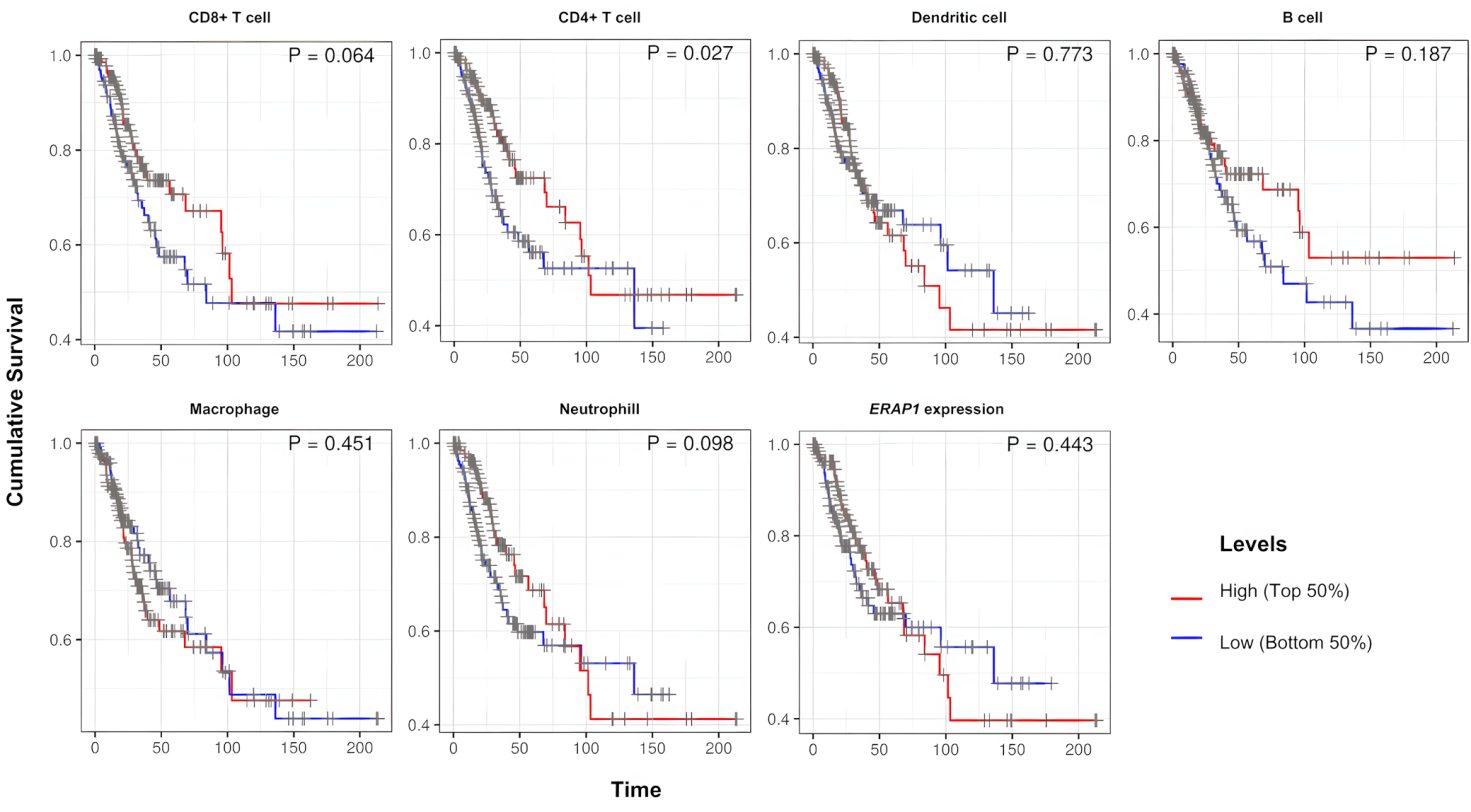
*ERAP1* expression and immune infiltration levels in cervical cancer patients retrieved from the TCGA‐CESC cohort. (A) Correlations between *ERAP1* expression and immune infiltration levels. *ERAP1* expression levels are shown in log2 TPM. The blue line represents the fitted trend, and the shaded area represents the confidence interval. (B) Kaplan–Meier survival curves for immune cell infiltrates and *ERAP1* expression in patients with cervical cancer. Survival curves are stratified by immune cell infiltration levels (B cells, CD8+ T cells, CD4+ T cells, macrophages, neutrophils, and dendritic cells) and *ERAP1* expression. Patients were divided into high (top 50%, red line) and low (bottom 50%, blue line) infiltration or expression groups. Log‐rank *p* values are shown for each comparison. The time to follow‐up was measured in months.

Multivariate Cox proportional hazards model was used to explore the effects of immune cell infiltrates (CD8+ T cells, CD4+ T cells, neutrophils, dendritic cells, macrophages, B cells), clinical variables (age and tumor stage), and *ERAP1* expression on survival (Table 7). CD8+ T cell infiltration was significantly associated with improved survival (HR = 0.006, *p* = 0.049), suggesting that higher levels of CD8+ T cell infiltration are associated with a reduced likelihood of death. In contrast, neutrophil (HR = 0.007, *p* = 0.314), macrophage (HR = 16.291, *p* = 0.448), dendritic (HR = 58.277, *p* = 0.102), and B cell (HR = 0.063, *p* = 0.558) infiltration did not reach statistical significance. Age (HR = 1.013, *p* = 0.137) and *ERAP1* expression (HR = 1.312, *p* = 0.079) were not significantly associated with survival.

**FIGURE 5:**
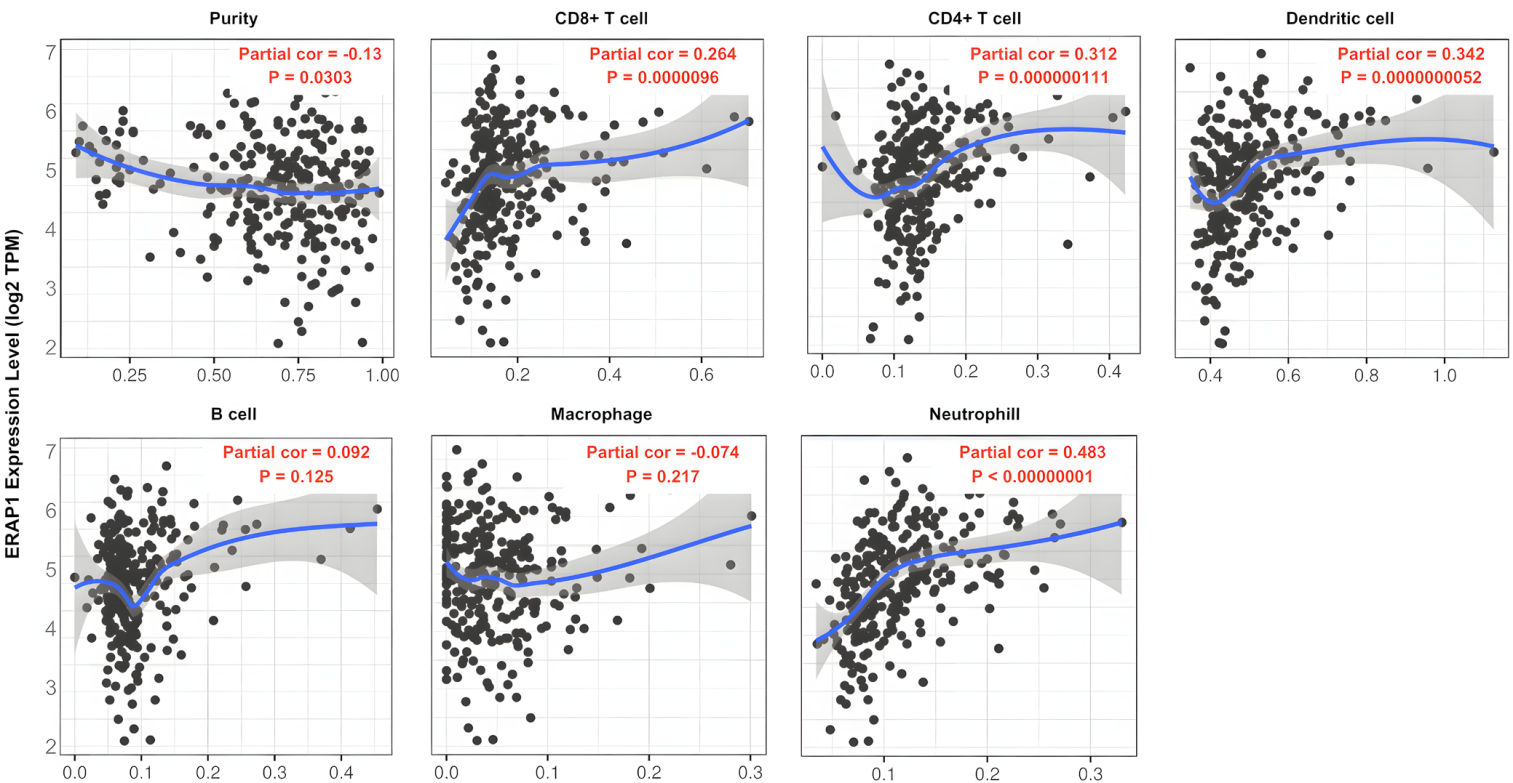
Correlation between ERAP1 expression and immune cell infiltration in cervical cancer patients from the TCGA‐CESC cohort. Scatter plots showing the partial correlation between *ERAP1* expression levels (log_2_ TPM) and tumor purity, CD8^+^ T cells, CD4^+^ T cells, dendritic cells, neutrophils, macrophages, and B cells in cervical cancer samples from the TCGA‐CESC cohort. Immune cell infiltration levels and tumor purity were estimated using The Tumor IMmune Estimation Resource (TIMER) tool. Correlations were assessed using partial Spearman's correlation adjusted for tumor purity, and the corresponding correlation coefficients and *p*‐values are shown in each panel.

Kaplan–Meier survival curves, which were used to assess survival differences over time based on immune cell infiltration and expression, supported some of these findings (Figure 4). Consistently, patients with greater CD8+ T cell infiltration showed a trend toward better survival (*p* = 0.064), whereas CD4+ T cell infiltration was significantly associated with improved survival (*p* = 0.027). Conversely, dendritic cell infiltration, macrophage infiltration, B‐cell infiltration, and *ERAP1* expression did not significantly affect survival (*p* = 0.773, *p* = 0.451, *p* = 0.187, and *p* = 0.443, respectively).

## 4 Discussion

The present study evaluated the impact of *ERAP1* genetic variants and haplotypes in two datasets of women diagnosed with cervical cancer to elucidate their role in prognosis across diverse populations. *ERAP1* encodes an endoplasmic reticulum aminopeptidase that processes peptides at the N‐terminus to facilitate binding to HLA‐I molecules. These HLA‐peptide complexes are subsequently displayed on the cell surface for recognition by cytotoxic CD8+ T cells, thus enabling immune surveillance and response (Țiburcă et al. 2024). Structurally, *ERAP1* protein comprises four domains (I–IV): domain I, which represents a structural sandwich domain; domain II, which serves as the protein's active site; domain III, which represents a β‐sandwich domain; and domain IV, which regulates enzymatic activity (Țiburcă et al. 2024). *ERAP1* operates in two conformational states: the open form, which facilitates substrate binding by exposing the catalytic site, and the closed form, optimized for peptide trimming (Li et al. 2024). Conformational changes, particularly in the active site of domain II, can influence the transition of *ERAP1* between the open and closed states, thereby affecting its enzymatic function (Hanson et al. 2019). *ERAP1* rs30187 (p.Lys528Arg) variant is located at the interface of domains II and III, where it can influence protein conformational dynamics and catalytic activity; therefore, allelic changes at this site may affect the conformational dynamics of *ERAP1* protein (Li et al. 2024). Additionally, p.Arg725Gln (rs17482078) and p.Gln730Glu (rs27044) variants have been associated with reduced protein activity and *ERAP1* expression, respectively, leading to a decrease in the expression of overall MHC class I genes (Reeves et al. 2013). Such alterations may result in suboptimal or excessive peptide triming, reshaping the immunopeptidome and potentially facilitating immune evasion in HPV‐driven tumors.

In this study, survival analysis of patients within the TCGA‐CESC dataset revealed significant associations between rs2287987 (p.Met349Val) and poorer clinical outcomes, with findings indicating an 11‐fold increased risk of overall mortality and a 13‐fold increased risk of cancer‐specific mortality. Kaplan–Meier survival curves demonstrated reduced overall and disease‐specific survival among individuals homozygous for this variant. Located near the catalytic site, rs2287987 impairs peptide trimming, which could reduce antigen presentation and weaken immune responses (Țiburcă et al. 2024). Previous studies corroborate these findings, suggesting a strong link between *ERAP1* variants and autoimmune disorders, such as ankylosing spondylitis (AS), Behçet's disease (BD), and psoriasis (Stratikos et al. 2014; Padula et al. 2020; Li et al. 2024; Lee and Song 2016; Zavattaro et al. 2020); however, no previous associations with cervical cancer have been reported. Moreover, Reeves et al. (2013) reported that alleles containing this variant alone exhibited an intermediate cleavage profile, with the capacity to generate mature epitopes depending on the N‐terminal amino acid of the peptide precursor. Together, this findings support the biological plausibility that rs2287987 affects the modulation of peptide trimming and HLA‐I‐restricted recognition.

Conversely, rs30187 (p.Lys528Arg) variant demonstrated a protective effect, with both heterozygous and homozygous variants linked to a reduced risk of mortality. Kaplan–Meier survival curves revealed a borderline association with overall survival, and no significant association was observed for disease‐specific survival. Located at the interface between domains II and III, rs30187 variant has been reported to be protective, reducing the risk of Behçet's disease by 4‐fold in individuals homozygous for this allele (Riahi et al. 2020); however, this variant was negatively associated with lymph node metastasis in cervical cancer patients (Mehta et al. 2017). Additionally, it is frequently associated with hypertension, a common symptom in patients with AS (Lee and Song 2016; Szczypiorska et al. 2011). In non‐HPV‐related tumors, Yang et al. (2021) revealed the association between rs30187 variant and high *ERAP1* mRNA expression levels, and low risk of death in patients with non‐small cell lung carcinoma (NSCLC). Wiśniewski et al. (2021) further reported significant association between *rs30187* and overall survival in patients with NSCLC, indicating an important role of *ERAP1* in the anticancer response. Moreover, although rs2287987 and rs30187 were individually associated with survival outcomes in the TCGA‐CESC dataset, this association was not retained in the multivariate Cox regression analysis, suggesting that these variants may not have an independent prognostic effect in this cohort. Although an association between these variants and clinical outcomes was not observed in the Brazilian patient cohort, the absence of statistical significance may be partly attributed to the limited sample size. Specifically, the cohort included only 52 Brazilian cervical cancer patients, which likely diminished the statistical power due to the small sample size and low number of events, thereby restricting the detection of modest effects tipocally expected for commom germline variants. Additionally, population‐specific differences, including genetic background and environmental factors, likely contribute to the observed discrepancy between cohorts. This finding suggests that the combination of variants may contribute to a better understanding of the impact of these variants on the prognosis of cervical cancer patients and the difference in association between studies, emphasizing the importance of considering population‐specific characteristics when evaluating biomarkers and understanding cervical cancer heterogeneity. Therefore, the lack of statistically significant associations in the Brazilian cohort does not exclude a biological role for *ERAP1* variants in cervical cancer prognosis, but rather underscores the need for larger, well‐powered validation studies in diverse populations to clarify their clinical relevance and capture population‐specific effects.

Arison of variant minor allele frequencies revealed distinct patterns between the TCGA, the Brazilian cohort, and the AbraOM database. Among the six variants evaluated in both cohorts, most showed consistent MAFs across datasets, including rs72773968 (p.Thr12Ile), rs10050860 (p.Asp575Asn), rs17482078 (p.Arg725Gln), and rs2287987 (p.Met349Val), suggesting shared genetic patterns. Significant variability was identified for rs30187 (p.Lys528Arg), which had a higher MAF in the Brazilian cohort and in ABraOM compared to the TCGA database. The high genetic variability of rs2287987 (p.Met349Val) and rs30187 (p.Lys528Arg) variants among the Brazilian population, both in healthy and cervical cancer individuals, may reflect the lack of significant association with cervical cancer prognosis in this population. Among the variants evaluated only in the TCGA cohort, rs3734016 (p.Glu56Lys), rs26653 (p.Arg127Pro), rs26618 (p.Ile276Met), and rs27895 (p.Gly346Asp) variants showed lower MAFs in the TCGA cohort compared to the AbraOM database, indicating potential representativeness of these variants in the Brazilian individuals even without direct data from the Brazilian cervical cancer cohort. This observation could imply that these *ERAP1* variants may not confer an increased risk for cervical cancer development but instead reflect the genetic background of the Brazilian population.

As individual variant analyses may not fully capture the complexity of ERAP1‐mediated effects, haplotype‐based analyses were performed in TCGA‐CESC patients based on tumor WES data. The cumulative effect of specific variant combinations may differ from that of individual variants (Li et al. 2024). Reeves et al. (Reeves et al. 2013) highlighted the polymorphic nature of *ERAP1* by identifying distinct haplotypes encoding alleles with unique abilities for cleaving amino acids at the N‐terminal portion of the substrate. *ERAP1* haplotype, comprising rs2287987 (p.Met349Val), rs30187 (p.Lys528Arg), rs10050860 (p.Asp575Asn), rs17482078 (p.Arg725Gln), and rs27044 (p.Gln730Glu) variants, was found to encode a nonfunctional protein, which may influence disease progression or survival. These variants are located in important catalytic sites and may lead to conformational changes that can influence the transition between the open and closed states, thereby affecting its enzymatic activity (Reeves et al. 2013). In the present study, patients homozygous for the H005 haplotype exhibited a markedly increased risk of mortality, whith a 9.5‐fold higher risk compared with heterozygous individuals and non‐carriers. This haplotype comprises the variants rs26653 (p.Arg127Pro), rs2287987 (p.Met349Val), rs30187 (p.Lys528Arg), rs17482078 (p.Arg725Gln), and rs27044 (p.Gln730Glu), overlapping substantially with the functionally impaired haplotype described by Reeves et al. (2013), and including three of these variants previously associated with worse cervical cancer prognosis (Mehta et al. 2009, 2007). Kaplan–Meier survival analysis confirmed that curves for patients homozygous for H005 diverged significantly from those representing heterozygous or noncarrier states. Notably, H005 haplotype included rs2287987 (p.Met349Val) variant, which was individually associated with poorer survival, and rs30187 (p.Lys528Arg) variant, which showed a protective association in single‐variant analyses. When evaluated within the haplotype context, however, the protective effect of rs30187 appeared to be attenuated, possibly reflecting complex interactions among *ERAP1* variants that collectively alter enzymatic activity and immune modulation. These findings indicate that haplotype‐based analyses provide a more biologically informative prognostic framework than single‐variant approaches by capturing the combined effects of multiple ERAP1 variants in antigen processing and immune responses.

Additionally, H023 haplotype was significantly associated with poorer overall survival, conferring a 15‐fold increased risk. Kaplan–Meier curves revealed distinct survival differences between carriers and non‐carriers of this haplotype. Although no individuals were homozygous for H023, the presence of a single copy was associated with reduced survival, indicating a strong prognostic effect in the heterozygous state. This haplotype comprises the rs26653 (p.Arg127Pro), rs27895 (p.Gly346Asp), rs30187 (p.Lys528Arg), and rs27044 (p.Gln730Glu) variants. Similar to H005, rs30187 (p.Lys528Arg) is included within H023, reinforcing the notion that the combined effect of these variants on peptide trimming can potentially amplify or counterbalance the effects of individual variants. Variants within this haplotype, including rs30187, are located in a domain junction region, which similarly affects the specificity or enzymatic activity of the aminopeptidase by influencing the conformational change between the enzyme's open and closed states (Stratikos et al. 2014; Padula et al. 2020). Consistent with this findings, Reeves et al. (2019) reported that *ERAP1* haplotypes carrying multiple variants, along with the rs30187 variant, exhibited poor trimming phenotypes, reflecting an intermediate cleavage profile, further supporting its potential impact on the enzymatic activity of *ERAP1*. Nevertheless, we strongly believe that these *ERAP1* haplotypes play a prognostic role in cervical cancer, as the combined presence of variants, particularly those identified in H005 and H023 haplotypes, may contribute to worse prognosis. The cumulative effect of these variants could alter *ERAP1* enzymatic activity, impairing antigen processing and HLA‐I‐mediated immune recognition, thereby potentially facilitating immune evasion in tumor cells. Future studies should focus on validating these haplotypes in the Brazilian population to confirm their prognostic relevance. Additionally, functional analyses will be conducted to further elucidate the biological mechanisms through which these haplotypes may influence tumor progression and patient outcomes.

With respect to clinicopathological characteristics, advanced‐stage disease (III/IV) was associated with poorer overall and disease‐specific survival among TCGA‐CESC patients, in agreement with previous studies. However, age distribution, tumor stage, and treatment modalities were not uniform across the analyzed cohorts, and these factors may have independently influenced survival outcomes, thereby potentially confounding associations with ERAP1 genetic variation. In the Brazilian cohort, the lack of association between tumor stage and survival is likely attributable to the small number of patients with advanced‐stage disease, which limits statistical power and underscores the challenges of comparing prognostic factors across cohorts with distinct clinical compositions.

*ERAP1* expression and its correlation with immune infiltration in cervical cancer suggest an active role of this gene in the tumor microenvironment, particularly in CD8+ and CD4+ T cell infiltration. The positive association between *ERAP1* expression and T cell infiltration, especially that of CD8+ and CD4+ T cells, suggests that *ERAP1* may modulate antitumor immune responses, promoting an environment conducive to the infiltration of effector immune cells. Moreover, the survival analysis results indicated that increased levels of CD8+ T cell infiltration are linked to improved survival, reinforcing the protective role of these cells in cervical cancer. When considered together, the association between *ERAP1* expression and CD8+ T‐cell infiltration suggests that *ERAP1* may contribute to a more immunologically active tumor microenvironment, potentially through optimized trimming of precursor peptides and enhanced HLA‐I–mediated antigen presentation, however, these findings should be interpreted as supportive associations rather than evidence of a direct causal relationship.

Regarding *HLA* alleles in the TCGA‐CESC cohort, HLA‐A*02:01 allele was observed in 32.95% of patients, notably higher than the global prevalence of 14.96%. Similarly, *HLA‐* B*07:02 and HLA‐C*07:02 alleles were more common in the cohort (23.12% and 28.32%, respectively) compared to their global rates (4.85% and 12.78%) (Robinson et al. 2020), likely reflecting regional and population‐dependent genetic diversity of *HLA* allele distribution. Previous studies have associated *HLA*‐B*07:02 and *HLA*‐C*07:02 with an increased risk of adenocarcinoma and cervical squamous cell carcinoma (Safaeian et al. 2014). Other alleles identified in this study have also been implicated in cervical cancer susceptibility, including *A*02:05, which has been linked to reduced risk, and *HLA*‐A*24:02 which has been associated with increased risk in the Asian population (Hosono et al. 2010). Although no direct association between *HLA‐I* genotypes and cervical cancer prognosis was identified in this study, the high prevalence of specific *HLA‐B* and *HLA‐C* alleles suggests that *HLA* background may contribute to disease risk and variability in immune responses. The lack of associations may be attributable to the extensive polymorphism of *HLA* genes and the limited sample size, highlighting the need for larger cohorts to further investigate these relationships.

## 5 Conclusions

We identified two *ERAP1* haplotypes, H005 and H023, that were independently associated with worse prognosis in TCGA‐CESC cervical cancer patients. Homozygosity for the H005 haplotype and the presence of H023 were significantly linked to a higher risk of both overall and disease‐specific mortality. Additionally, *ERAP1* variants rs2287987 and rs30187 were individually associated with survival; however, these associations were not retained in multivariate analysis and were not observed in the Brazilian cervical cancer cohort. In contrast, increased levels of CD8^+^ T cell infiltration were associated with improved survival, reinforcing the protective role of these immune cells in cervical cancer. The analysis of two independent cervical cancer cohorts, including the TCGA‐CESC dataset and a cohort of Brazilian patients, allowed for the evaluation of *ERAP1* variants across different genetic backgrounds. This comparative approach provided valuable insights into their potential clinical relevance and underscored the importance of assessing these variants at the haplotype level rather than as isolated variants.

In parallel, increased CD8+ T‐cell infiltration was consistently associated with improved survival, reinforcing the central role of antitumor immune responses in cervical cancer and supporting a functional link between *ERAP1*‐mediated antigen processing and immune surveillance. The comparative analysis of two independent cohorts with distinct genetic backgrounds highlighted both the potential prognostic relevance of *ERAP1* haplotypes and the influence of population‐specific factors on genetic associations. Future research should prioritize validating the prognostic significance of H005 and H023 haplotypes, as well as CD8^+^ T cell infiltration levels, in independent cohorts, including the Brazilian population, to assess their reproducibility across diverse genetic backgrounds. Additionally, functional studies are crucial to elucidate the molecular mechanisms through which these haplotypes influence *ERAP1* enzymatic activity, antigen processing, and immune evasion in cervical cancer. Establishing the prognostic role of these haplotypes could enhance risk stratification, enabling the identification of patients at higher risk of poor survival.

### 5.1 Study Limitations

The lack of haplotype identification in samples from the Brazilian cervical cancer dataset hinders the analysis of *ERAP1* at the haplotype level within this population. Future investigations should prioritize the inclusion of haplotype identification methods to thoroughly evaluate their associations with clinical outcomes. Specifically, assessing the association of haplotypes identified in silico, such as H005 and H023, within this population could offer valuable insights into the genetic factors that influence cervical cancer outcomes and the significance of *ERAP1* haplotypes in cervical cancer prognosis.

## Author Contributions

Ana Luisa Dian: Conceptualization (lead), formal analysis (lead), methodology (lead), writing – original draft (lead), and writing – review and editing (equal). Ana Paula Almeida Cunha: Conceptualization (lead), formal analysis (lead), methodology (lead), writing – original draft (lead), and writing – review and editing (lead). Carolina Pereira de Souza Melo: Conceptualization (equal), formal analysis (equal), and methodology (equal). Maria do Desterro Soares Brandão Nascimento: Resources (equal). Maria Gabriela Reis Carvalho: Funding acquisition (equal), resources (equal), supervision (equal), and writing – review and editing (equal). Jeronimo Conceição Ruiz: Conceptualization (equal), formal analysis (equal), funding acquisition (equal), methodology (equal), supervision (equal), and writing – review and editing (equal).

## Funding

Conselho Nacional de Desenvolvimento Científico e Tecnológico (CNPq, grant: 313108/2022‐6); Fundação de Amparo à Pesquisa do Estado de Minas Gerais (FAPEMIG Network, grant: RED‐00104‐22); Coordenação de Aperfeiçoamento de Pessoal de Nível Superior (CAPES); Fundação de Amparo à Pesquisa do Estado do Rio de Janeiro (FAPERJ). The funders had no role in the study design, data collection and analysis, decision to publish, or manuscript preparation.

## Ethics Statement

The study was approved by the Ethical Review Board of the René Rachou Institute/Fiocruz (approval number 5.534.968), according to the 466/2012 Resolution of the National Board of Health.

## Consent

All participants submitted written informed consent forms before data collection.

## Supporting information


**Appendix S1:** mgg370170‐sup‐0001‐AppendixS1.pdf.

## Data Availability

The WES data used in this study were obtained from The Cancer Genome Atlas Cervical Squamous Cell Carcinoma and Endocervical Adenocarcinoma (TCGA‐CESC) and were deposited in the Genomic Data Commons (GDC) Data Portal of the National Institute of Health (NIH) (https://gdc‐portal.nci.nih.gov/) under the accession code: phs000178.v9.p8. *ERAP1* sequencing data for the cohort of Brazilian patients with cervical cancer are available upon request.
