# Supplementary material for: Prognostic Impact of ERAP1 Genetic Variants and Haplotypes in Cervical Cancer: Insights From TCGA‐CESC and a Brazilian Patient Cohort
Source: Mol Genet Genomic Med. 2026 Oct 20;14(10):e70170. doi: 10.1002/mgg3.70170 (PMC13681744; doi:10.1002/mgg3.70170)
Supplement: Supplementary file 1 [file MGG3-14-e70170-s001.pdf]

RESEARCH ARTICLE:

Title: Prognostic impact of *ERAP1* genetic variants and haplotypes in cervical cancer: Insights from TCGA-CESC and a Brazilian patient cohort

Running title: Impact of *ERAP1* variants on cervical cancer survival

Supplementary information regarding sample information and association analyses between *ERAP1* variants and cervical cancer

Figure S1. Identification of *ERAP1* variants and determination of haplotypes. Correlation of allele frequencies (AF) of *ERAP1* SNPs from the reference panel with the AF of *ERAP1* SNPs from WES data from **(A)** blood and **(B)** tumor tissue from TCGA patients. Nearly all variant AFs from the 173 cervical cancer patients' data are positively correlated with variant AFs from the reference panel, and the data show a smooth, narrow diagonal line. Outliers correspond to polymorphisms located in introns, and therefore not included in the study. **C)** Distribution of *ERAP1* missense variants in whole-exome sequencing (WES) of blood (black) and tumor (red) samples. **D)** The 10 most frequently detected *ERAP1* haplotypes in the TCGA dataset. The observations correspond to the number of times the haplotype was detected, counting once for heterozygous patients and twice for homozygous patients. **E)** The most frequently detected *ERAP1* haplotype combinations in the TCGA dataset.

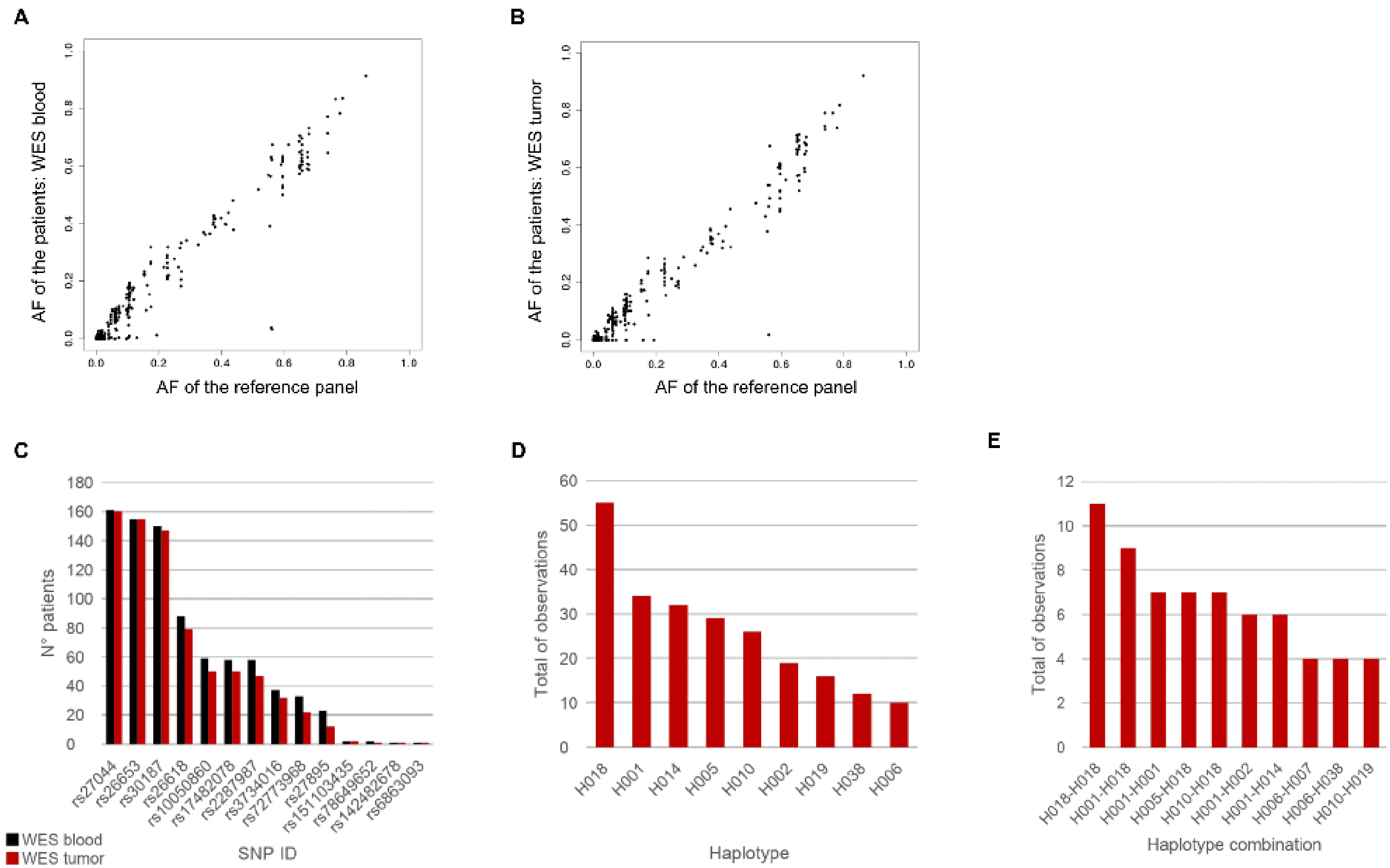

Figure S2. Distribution of HLA I alleles at the HLA-A, HLA-B, and HLA-C *loci* among 173 cervical cancer patients from the TCGA-CESC dataset.

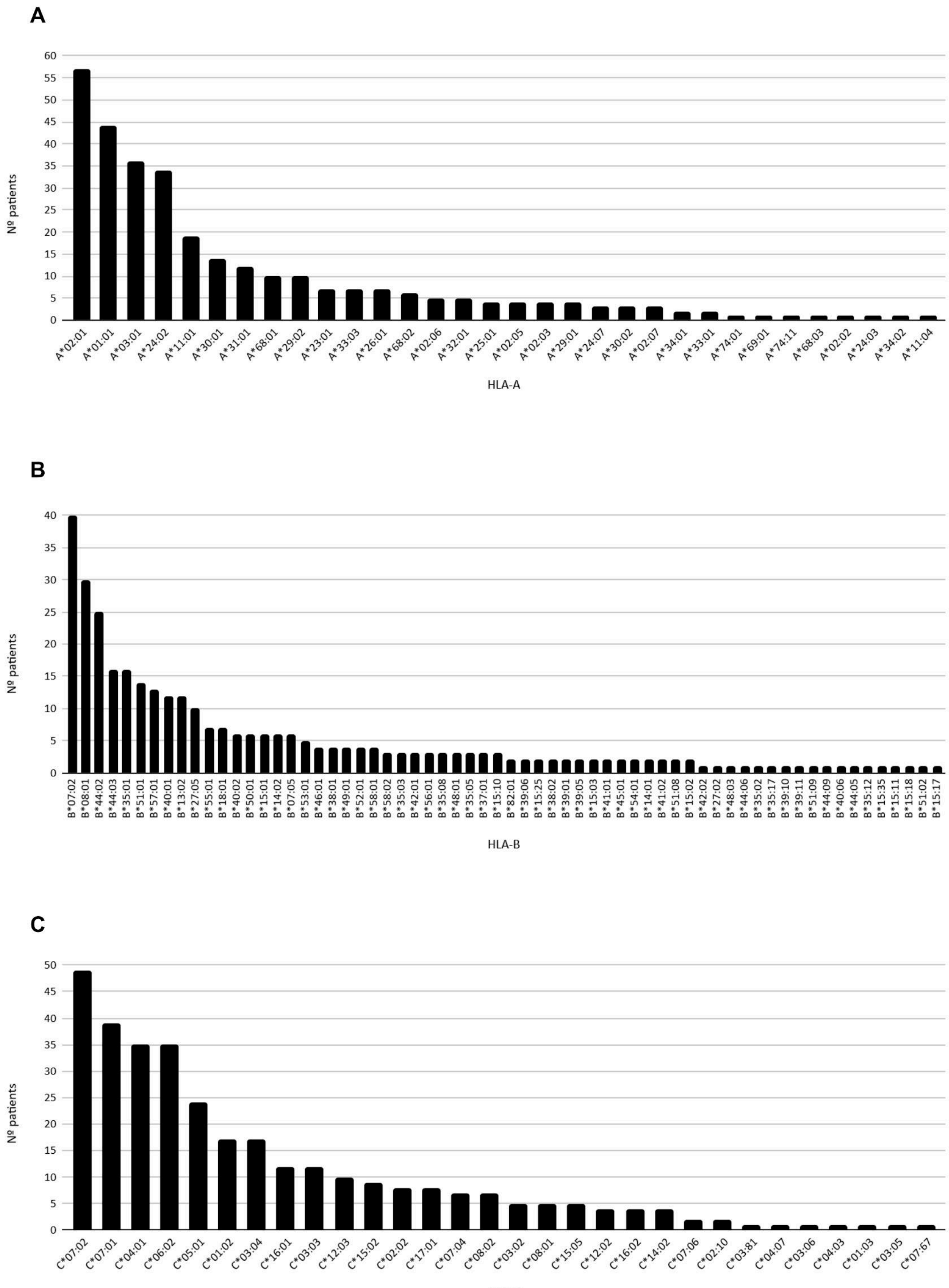

Table S1. Clinicopathological data of 173 patients diagnosed with cervical cancer from the TCGA-CESC dataset.

Clinicopathological data		N	%
**FIGO staging**	NA	3	1.68
	I	91	51.12
	II	28	15.73
	III/IV	56	31.46
**Histological type of cancer**	Squamous cell carcinoma	144	80.9
	Adenosquamous carcinoma	3	1.68
	Adenocarcinoma	31	17.42
**HPV**	Present	169	94.94
	Absent	9	5.06
**Age at diagnosis**	Under 49 years	101	58.38
	Over 49 years	72	41.62
**Lymphovascular invasion**	Present	61	34.27
	Absent	59	33.15
	NA	58	32.58
**Lymph nodes metastasis**	Present	38	21.35
	Absent	98	55.06
	NA	42	23.60

NA = no data; FIGO = disease staging, with FIGO I being the lowest degree of disease dissemination and FIGO III/IV the highest degree.

Table S2. Characteristics of the Brazilian cervical cancer patients included in this study (N = 52).

Clinicopathological data		N	%
**FIGO staging**	I	8	15.38
	II	17	32.69
	III/IV	27	51.92
**Histological type of cancer**	Squamous cell carcinoma	45	86.54
	Adenocarcinoma	5	9.62
	NA	2	3.85
**HPV**	Present	44	84.62
	Absent	8	15.38
**Age at diagnosis**	Under 49 years	27	51.92
	Over 49 years	25	48.08
**Lymphovascular invasion**	Present	5	9.62
	Absent	13	25.00
	NA	34	65.38
**Lymph nodes metastasis**	Present	4	7.69
	Absent	13	25.00
	NA	35	67.31

NA = no data; FIGO = disease staging, with FIGO I being the lowest degree of disease dissemination and FIGO III_IV the highest degree.

Table S3. Average coverage values for each *ERAP1* variant identified in the TCGA-CESC dataset.

Variant	Coverage: median (interval)	
	WES blood	WES tumor
rs72773968 (p.Arg127Pro)	34x (14 - 92x)	35x (14 - 91x)
rs3734016 (p.Glu56Lys)	83x (44 - 154x)	81x (27 - 146x)
rs26653 (p.Arg127Pro)	77x (34 - 170x)	76x (30 - 155x)
rs26618 (p.Ile276Met)	68x (19 - 140x)	63x (16-130)
rs27895 (p.Gly346Asp)	48x (20 - 85x)	46x (15 - 91x)
rs2287987 (p.Met349Val)	51x (22 - 95x)	48x (14 - 96x)
rs30187 (p.Lys528Arg)	56x (26 - 100x)	58x (12 - 112x)
rs10050860 (p.Asp575Asn)	39x (12 - 103x)	39x (15 - 99x)
rs17482078 (p.Arg725Gln)	76x (32 - 300x)	77x (25 - 277x)
rs27044 (p.Gln730Glu)	74x (32 - 273x)	76x (24 - 263x)

Table S4: Primer sequences for the identification of *ERAP1* variants among the Brazilian dataset of cervical cancer patients.

Target Name	ERAP1 Variant	Primer Sequence (5'-3')	Product length (bps)	PCR condition	Position in the genome (GRCh38)	Alleles
p.Thr12Ile	rs72773968	*Forward:*<br>*GCTGACTGGCTGTGATTT*<br>*Reverse:*<br>*GCTTGTTCGGTTGCTTTC*	571	Initial denaturation: 94ºC / 2 min<br>Number of cycles: 35<br>Denaturation: 94°C / 30 s<br>Annealing: °C / 30 s<br>Elongation: 72°C / 2 min	5: 96803892	G>A,T
p.Met349Val	rs2287987	*Forward:*<br>*AGCCTTATATGTCCCATAGAAGCAA TA*<br>*Reverse:*<br>*CCGACTTTCAGTCTGGTGCT*	211	Initial denaturation: 94ºC / 2 min<br>Number of cycles: 35<br>Denaturation: 94°C / 30 s<br>Annealing: 56°C / 30 s<br>Elongation: 72°C / 2 min	5: 96793832	T>A,C
p.Lys528Arg	rs30187	*Forward:*<br>*GGCAGATGTTACCTAGACAACA*<br>*Reverse:*<br>*ATTGGCATCAGGAAGGGGTG*	203	Initial denaturation: 94ºC / 2 min<br>Number of cycles: 35<br>Denaturation: 94°C / 30 s<br>Annealing: 56°C / 30 s<br>Elongation: 72°C / 2 min	5: 96788627	T>A,C
p.Asp575Asn	rs10050860	*Forward:*<br>*TCCTACACATTTTCACATTCCTCCT*<br>*Reverse:*<br>*TGCCTGGCACATGATAGGTG*	219	Initial denaturation: 94ºC / 2 min<br>Number of cycles: 35<br>Denaturation: 94°C / 30 s<br>Annealing: 57°C / 30 s<br>Elongation: 72°C / 2 min	5: 96786506	C>T
p.Arg725Gln	rs17482078	*Forward:*<br>*GGACTGACCTCAAGTTTCCA*<br>*Reverse:*<br>*TTTCCCTGTACAACGCCCTC*	215	Initial denaturation: 94ºC / 2 min<br>Number of cycles: 35<br>Denaturation: 94°C / 30 s<br>Annealing: 56°C / 30 s<br>Elongation: 72°C / 2 min	5: 96783162<br>5: 96783148	C>G,T
p.Gln730Glu	rs27044					G>A,C

Table S5. Summary of ERAP1 haplotypes identified among the cervical cancer patients from the TCGA-CESC dataset (according to GRCh38 human reference genome).

Haplotype ID	ERAP1 Single Nucleotide Polymorphism (SNP) position in chromosome 5 (GRCh38)									
	96803892	96803761	96803547	96795133	96793840	96793832	96788627	96786506	96783162	96783148
H001	-	-	-	-	-	-	-	-	-	-
H002	rs72773968	-	rs26653	-	-	-	-	-	-	-
H003	rs72773968	-	rs26653	-	-	-	-	-	rs17482078	rs27044
H004	-	-	rs26653	-	-	rs2287987	rs30187	rs10050860	-	-
H005	-	-	rs26653	-	-	rs2287987	rs30187	rs10050860	rs17482078	rs27044
H006	-	-	-	rs26618	-	-	rs30187	-	-	rs27044
H007	-	-	rs26653	-	-	-	-	-	-	-
H008	-	-	-	-	-	-	rs30187	-	-	rs27044
H009	-	-	rs26653	rs26618	-	-	-	-	-	rs27044
H010	-	-	rs26653	-	-	-	rs30187	-	-	rs27044
H011	-	-	rs26653	-	-	rs2287987	rs30187	-	rs17482078	rs27044
H012	-	-	rs26653	-	-	-	rs30187	rs10050860	-	rs27044
H013	-	-	rs26653	-	-	rs2287987	rs30187	rs10050860	-	rs27044
H014	-	-	-	-	-	-	-	-	-	rs27044
H015	-	-	rs26653	-	-	rs2287987	-	-	rs17482078	-
H016	-	-	rs26653	rs26618	-	-	rs30187	rs10050860	-	rs27044
H017	-	-	-	-	rs27895	-	rs30187	-	-	-
H018	-	-	rs26653	rs26618	-	-	rs30187	-	-	rs27044
H019	-	rs3734016	rs26653	-	-	-	rs30187	-	-	rs27044
H020	-	-	-	-	rs27895	-	rs30187	-	-	rs27044
H021	rs72773968	-	rs26653	-	-	-	-	-	-	rs27044
H022	-	-	-	-	-	-	-	-	rs17482078	rs27044
H023	-	-	rs26653	-	rs27895	-	rs30187	-	-	rs27044
H024	-	-	rs26653	-	rs27895	-	rs30187	-	-	-
H025	-	rs3734016	rs26653	rs26618	-	-	rs30187	-	-	rs27044
H026	-	-	rs26653	-	-	-	rs30187	rs10050860	rs17482078	rs27044
H027	-	-	rs26653	rs26618	-	-	-	-	rs17482078	rs27044
H028	rs72773968	-	rs26653	-	-	-	rs30187	rs10050860	-	rs27044
H029	-	-	-	-	-	-	-	-	rs17482078	-
H030	-	-	rs26653	-	-	-	rs30187	-	-	-
H031	-	-	-	rs26618	-	-	-	-	-	rs27044
H032	-	-	rs26653	rs26618	-	-	rs30187	-	-	-
H033	-	-	rs26653	-	-	-	rs30187	-	rs17482078	rs27044
H034	rs72773968	-	rs26653	-	-	-	rs30187	-	-	rs27044

**H035**	-	-	rs26653	-	-	rs2287987	-	rs10050860	rs17482078	-
**H036**	-	-	rs26653	rs26618	-	-	rs30187	-	rs17482078	rs27044
**H037**	rs72773968	-	rs26653	rs26618	-	-	rs30187	-	-	rs27044
**H038**	-	-	rs26653	-	-	-	-	-	-	rs27044
**H039**	-	rs3734016	rs26653	-	-	-	-	-	-	-
**H040**	-	-	-	rs26618	-	-	rs30187	-	-	-
**H041**	rs72773968	-	rs26653	rs26618	-	-	rs30187	-	-	-
**H042**	-	-	rs26653	-	-	-	-	-	rs17482078	rs27044
**H043**	-	-	-	-	-	rs2287987	rs30187	rs10050860	-	-
**H044**	rs72773968	-	rs26653	-	-	-	rs30187	-	-	-
**H045**	-	rs3734016	rs26653	-	-	-	-	-	-	rs27044
**H046**	-	-	-	-	-	-	rs30187	-	-	-
**H047**	-	-	-	-	-	rs2287987	rs30187	rs10050860	rs17482078	rs27044
**H048**	-	-	-	-	-	rs2287987	rs30187	rs10050860	-	rs27044
**H049**	-	-	rs26653	-	-	rs2287987	rs30187	rs10050860	rs17482078	-
**H050**	-	-	-	-	rs27895	rs2287987	rs30187	rs10050860	rs17482078	rs27044
**H051**	-	-	rs26653	-	-	rs2287987	rs30187	-	-	rs27044
**H052**	-	rs3734016	rs26653	-	-	-	rs30187	-	-	-

Table S6. Haplotype combination of cervical cancer patients among the TCGA-CESC dataset.

			Amino Acid Position in *ERAP1*									
			12	56	127	276	346	349	528	575	725	730
**Alelles**	**Pacient**	**Haplotype**	T	E	R	I	G	M	K	D	R	Q
1	TCGA-BI-A0VR	1	T	E	R	I	G	M	K	D	R	Q
2		2	**I**	E	**P**	I	G	M	K	D	R	Q
1	TCGA-BI-A0VS	3	**I**	E	**P**	I	G	M	K	D	**Q**	**E**
2		4	T	E	**P**	I	G	**V**	**R**	**N**	R	Q
1	TCGA-BI-A20A	2	**I**	E	**P**	I	G	M	K	D	R	Q
2		5	T	E	**P**	I	G	**V**	**R**	**N**	**Q**	**E**
1	TCGA-C5-A1BE	6	T	E	R	**M**	G	M	**R**	D	R	**E**
2		7	T	E	**P**	I	G	M	K	D	R	Q
1	TCGA-C5-A1BF	5	T	E	**P**	I	G	**V**	**R**	**N**	**Q**	**E**
2		5	T	E	**P**	I	G	**V**	**R**	**N**	**Q**	**E**
1	TCGA-C5-A1BI	6	T	E	R	**M**	G	M	**R**	D	R	**E**
2		7	T	E	**P**	I	G	M	K	D	R	Q
1	TCGA-C5-A1BJ	8	T	E	R	I	G	M	**R**	D	R	**E**
2		9	T	E	**P**	**M**	G	M	K	D	R	**E**
1	TCGA-C5-A1BK	10	T	E	**P**	I	G	M	**R**	D	R	**E**
2		8	T	E	R	I	G	M	**R**	D	R	**E**
1	TCGA-C5-A1BL	11	T	E	**P**	I	G	**V**	**R**	D	**Q**	**E**
2		12	T	E	**P**	I	G	M	**R**	**N**	R	**E**
1	TCGA-C5-A1BM	13	T	E	**P**	I	G	**V**	**R**	**N**	R	**E**
2		14	T	E	R	I	G	M	K	D	R	**E**
1	TCGA-C5-A1BN	5	T	E	**P**	I	G	**V**	**R**	**N**	**Q**	**E**
2		5	T	E	**P**	I	G	**V**	**R**	**N**	**Q**	**E**
1	TCGA-C5-A1BQ	15	T	E	**P**	I	G	**V**	K	D	**Q**	Q
2		16	T	E	**P**	**M**	G	M	**R**	**N**	R	**E**
1	TCGA-C5-A1M5	17	T	E	R	I	**D**	M	**R**	D	R	Q

2		14	T	E	R	I	G	M	K	D	R	**E**
1	TCGA-C5-A1M6	2	**I**	E	**P**	I	G	M	K	D	R	Q
2		14	T	E	R	I	G	M	K	D	R	**E**
1	TCGA-C5-A1M7	5	T	E	**P**	I	G	**V**	**R**	**N**	**Q**	**E**
2		14	T	E	R	I	G	M	K	D	R	**E**
1	TCGA-C5-A1M8	18	T	E	**P**	**M**	G	M	**R**	D	R	**E**
2		18	T	E	**P**	**M**	G	M	**R**	D	R	**E**
1	TCGA-C5-A1M9	5	T	E	**P**	I	G	**V**	**R**	**N**	**Q**	**E**
2		10	T	E	**P**	I	G	M	**R**	D	R	**E**
1	TCGA-C5-A1ME	19	T	**K**	**P**	I	G	M	**R**	D	R	**E**
2		20	T	E	R	I	**D**	M	**R**	D	R	**E**
1	TCGA-C5-A1MF	5	T	E	**P**	I	G	**V**	**R**	**N**	**Q**	**E**
2		19	T	**K**	**P**	I	G	M	**R**	D	R	**E**
1	TCGA-C5-A1MH	21	**I**	E	**P**	I	G	M	K	D	R	**E**
2		1	T	E	R	I	G	M	K	D	R	Q
1	TCGA-C5-A1MI	22	T	E	R	I	G	M	K	D	**Q**	**E**
2		4	T	E	**P**	I	G	**V**	**R**	**N**	R	Q
1	TCGA-C5-A1MJ	10	T	E	**P**	I	G	M	**R**	D	R	**E**
2		5	T	E	**P**	I	G	**V**	**R**	**N**	**Q**	**E**
1	TCGA-C5-A1MK	2	**I**	E	**P**	I	G	M	K	D	R	Q
2		1	T	E	R	I	G	M	K	D	R	Q
1	TCGA-C5-A1ML	18	T	E	**P**	**M**	G	M	**R**	D	R	**E**
2		1	T	E	R	I	G	M	K	D	R	Q
1	TCGA-C5-A1MN	18	T	E	**P**	**M**	G	M	**R**	D	R	**E**
2		18	T	E	**P**	**M**	G	M	**R**	D	R	**E**
1	TCGA-C5-A1MP	23	T	E	**P**	I	**D**	M	**R**	D	R	**E**
2		14	T	E	R	I	G	M	K	D	R	**E**
1	TCGA-C5-A2LS	10	T	E	**P**	I	G	M	**R**	D	R	**E**
2		18	T	E	**P**	**M**	G	M	**R**	D	R	**E**
1	TCGA-C5-A2LT	10	T	E	**P**	I	G	M	**R**	D	R	**E**

2		5	T	E	**P**	I	G	**V**	**R**	**N**	**Q**	**E**
1	TCGA-C5-A2LV	24	T	E	**P**	I	**D**	M	**R**	D	R	Q
2		14	T	E	R	I	G	M	K	D	R	**E**
1	TCGA-C5-A2LX	18	T	E	**P**	**M**	G	M	**R**	D	R	**E**
2		19	T	**K**	**P**	I	G	M	**R**	D	R	**E**
1	TCGA-C5-A2LY	25	T	**K**	**P**	**M**	G	M	**R**	D	R	**E**
2		10	T	E	**P**	I	G	M	**R**	D	R	**E**
1	TCGA-C5-A2LZ	10	T	E	**P**	I	G	M	**R**	D	R	**E**
2		2	**I**	E	**P**	I	G	M	K	D	R	Q
1	TCGA-C5-A2M1	18	T	E	**P**	**M**	G	M	**R**	D	R	**E**
2		10	T	E	**P**	I	G	M	**R**	D	R	**E**
1	TCGA-C5-A2M2	1	T	E	R	I	G	M	K	D	R	Q
2		14	T	E	R	I	G	M	K	D	R	**E**
1	TCGA-C5-A3HD	5	T	E	**P**	I	G	**V**	**R**	**N**	**Q**	**E**
2		26	T	E	**P**	I	G	M	**R**	**N**	**Q**	**E**
1	TCGA-C5-A3HE	27	T	E	**P**	**M**	G	M	K	D	**Q**	**E**
2		13	T	E	**P**	I	G	**V**	**R**	**N**	R	**E**
1	TCGA-C5-A3HF	1	T	E	R	I	G	M	K	D	R	Q
2		21	**I**	E	**P**	I	G	M	K	D	R	**E**
1	TCGA-C5-A3HL	28	**I**	E	**P**	I	G	M	**R**	**N**	R	**E**
2		29	T	E	R	I	G	M	K	D	**Q**	Q
1	TCGA-C5-A7CG	30	T	E	**P**	I	G	M	**R**	D	R	Q
2		14	T	E	R	I	G	M	K	D	R	**E**
1	TCGA-C5-A7CH	5	T	E	**P**	I	G	**V**	**R**	**N**	**Q**	**E**
2		26	T	E	**P**	I	G	M	**R**	**N**	**Q**	**E**
1	TCGA-C5-A7CJ	2	**I**	E	**P**	I	G	M	K	D	R	Q
2		1	T	E	R	I	G	M	K	D	R	Q
1	TCGA-C5-A7CK	10	T	E	**P**	I	G	M	**R**	D	R	**E**
2		19	T	**K**	**P**	I	G	M	**R**	D	R	**E**
1	TCGA-C5-A7CL	5	T	E	**P**	I	G	**V**	**R**	**N**	**Q**	**E**

2		18	T	E	P	M	G	M	R	D	R	E
1	TCGA-C5-A7CM	31	T	E	R	M	G	M	K	D	R	E
2		10	T	E	P	I	G	M	R	D	R	E
1	TCGA-C5-A7UE	21	I	E	P	I	G	M	K	D	R	E
2		32	T	E	P	M	G	M	R	D	R	Q
1	TCGA-DG-A2KH	18	T	E	P	M	G	M	R	D	R	E
2		2	I	E	P	I	G	M	K	D	R	Q
1	TCGA-DG-A2KJ	14	T	E	R	I	G	M	K	D	R	E
2		18	T	E	P	M	G	M	R	D	R	E
1	TCGA-DG-A2KK	13	T	E	P	I	G	V	R	N	R	E
2		33	T	E	P	I	G	M	R	D	Q	E
1	TCGA-DG-A2KL	34	I	E	P	I	G	M	R	D	R	E
2		35	T	E	P	I	G	V	K	N	Q	Q
1	TCGA-DG-A2KM	14	T	E	R	I	G	M	K	D	R	E
2		18	T	E	P	M	G	M	R	D	R	E
1	TCGA-DR-A0ZL	19	T	K	P	I	G	M	R	D	R	E
2		18	T	E	P	M	G	M	R	D	R	E
1	TCGA-DR-A0ZM	2	I	E	P	I	G	M	K	D	R	Q
2		5	T	E	P	I	G	V	R	N	Q	E
1	TCGA-DS-A0VK	18	T	E	P	M	G	M	R	D	R	E
2		14	T	E	R	I	G	M	K	D	R	E
1	TCGA-DS-A0VL	19	T	K	P	I	G	M	R	D	R	E
2		1	T	E	R	I	G	M	K	D	R	Q
1	TCGA-DS-A0VM	12	T	E	P	I	G	M	R	N	R	E
2		36	T	E	P	M	G	M	R	D	Q	E
1	TCGA-DS-A0VN	18	T	E	P	M	G	M	R	D	R	E
2		1	T	E	R	I	G	M	K	D	R	Q
1	TCGA-DS-A3LQ	18	T	E	P	M	G	M	R	D	R	E
2		18	T	E	P	M	G	M	R	D	R	E
1	TCGA-DS-A5RQ	14	T	E	R	I	G	M	K	D	R	E

2		14	T	E	R	I	G	M	K	D	R	**E**
1	TCGA-EA-A1QS	10	T	E	**P**	I	G	M	**R**	D	R	**E**
2		10	T	E	**P**	I	G	M	**R**	D	R	**E**
1	TCGA-EA-A1QT	18	T	E	**P**	**M**	G	M	**R**	D	R	**E**
2		36	T	E	**P**	**M**	G	M	**R**	D	**Q**	**E**
1	TCGA-EA-A3HQ	5	T	E	**P**	I	G	**V**	**R**	**N**	**Q**	**E**
2		5	T	E	**P**	I	G	**V**	**R**	**N**	**Q**	**E**
1	TCGA-EA-A3HR	33	T	E	**P**	I	G	M	**R**	D	**Q**	**E**
2		18	T	E	**P**	**M**	G	M	**R**	D	R	**E**
1	TCGA-EA-A3HT	37	**I**	E	**P**	**M**	G	M	**R**	D	R	**E**
2		7	T	E	**P**	I	G	M	K	D	R	Q
1	TCGA-EA-A3HU	1	T	E	R	I	G	M	K	D	R	Q
2		18	T	E	**P**	**M**	G	M	**R**	D	R	**E**
1	TCGA-EA-A3QD	6	T	E	R	**M**	G	M	**R**	D	R	**E**
2		38	T	E	**P**	I	G	M	K	D	R	**E**
1	TCGA-EA-A3QE	19	T	**K**	**P**	I	G	M	**R**	D	R	**E**
2		5	T	E	**P**	I	G	**V**	**R**	**N**	**Q**	**E**
1	TCGA-EA-A3Y4	1	T	E	R	I	G	M	K	D	R	Q
2		14	T	E	R	I	G	M	K	D	R	**E**
1	TCGA-EA-A410	13	T	E	**P**	I	G	**V**	**R**	**N**	R	**E**
2		36	T	E	**P**	**M**	G	M	**R**	D	**Q**	**E**
1	TCGA-EA-A411	13	T	E	**P**	I	G	**V**	**R**	**N**	R	**E**
2		36	T	E	**P**	**M**	G	M	**R**	D	**Q**	**E**
1	TCGA-EA-A439	39	T	**K**	**P**	I	G	M	K	D	R	Q
2		8	T	E	R	I	G	M	**R**	D	R	**E**
1	TCGA-EA-A43B	5	T	E	**P**	I	G	**V**	**R**	**N**	**Q**	**E**
2		1	T	E	R	I	G	M	K	D	R	Q
1	TCGA-EA-A44S	5	T	E	**P**	I	G	**V**	**R**	**N**	**Q**	**E**
2		18	T	E	**P**	**M**	G	M	**R**	D	R	**E**
1	TCGA-EA-A4BA	10	T	E	**P**	I	G	M	**R**	D	R	**E**

2		1	T	E	R	I	G	M	K	D	R	Q
1	TCGA-EA-A50E	18	T	E	**P**	**M**	G	M	**R**	D	R	**E**
2		10	T	E	**P**	I	G	M	**R**	D	R	**E**
1	TCGA-EA-A556	39	T	**K**	**P**	I	G	M	K	D	R	Q
2		8	T	E	R	I	G	M	**R**	D	R	**E**
1	TCGA-EA-A5FO	13	T	E	**P**	I	G	**V**	**R**	**N**	R	**E**
2		36	T	E	**P**	**M**	G	M	**R**	D	**Q**	**E**
1	TCGA-EA-A5O9	18	T	E	**P**	**M**	G	M	**R**	D	R	**E**
2		18	T	E	**P**	**M**	G	M	**R**	D	R	**E**
1	TCGA-EA-A5ZD	13	T	E	**P**	I	G	**V**	**R**	**N**	R	**E**
2		36	T	E	**P**	**M**	G	M	**R**	D	**Q**	**E**
1	TCGA-EA-A5ZE	40	T	E	R	**M**	G	M	**R**	D	R	Q
2		38	T	E	**P**	I	G	M	K	D	R	**E**
1	TCGA-EA-A5ZF	10	T	E	**P**	I	G	M	**R**	D	R	**E**
2		19	T	**K**	**P**	I	G	M	**R**	D	R	**E**
1	TCGA-EA-A6QX	1	T	E	R	I	G	M	K	D	R	Q
2		18	T	E	**P**	**M**	G	M	**R**	D	R	**E**
1	TCGA-EA-A78R	14	T	E	R	I	G	M	K	D	R	**E**
2		32	T	E	**P**	**M**	G	M	**R**	D	R	Q
1	TCGA-EK-A2GZ	17	T	E	R	I	**D**	M	**R**	D	R	Q
2		14	T	E	R	I	G	M	K	D	R	**E**
1	TCGA-EK-A2H0	41	**I**	E	**P**	**M**	G	M	**R**	D	R	Q
2		38	T	E	**P**	I	G	M	K	D	R	**E**
1	TCGA-EK-A2H1	14	T	E	R	I	G	M	K	D	R	**E**
2		10	T	E	**P**	I	G	M	**R**	D	R	**E**
1	TCGA-EK-A2IP	2	**I**	E	**P**	I	G	M	K	D	R	Q
2		13	T	E	**P**	I	G	**V**	**R**	**N**	R	**E**
1	TCGA-EK-A2PI	10	T	E	**P**	I	G	M	**R**	D	R	**E**
2		1	T	E	R	I	G	M	K	D	R	Q
1	TCGA-EK-A2PK	42	T	E	**P**	I	G	M	K	D	**Q**	**E**

2		43	T	E	R	I	G	**V**	**R**	**N**	R	Q
1	TCGA-EK-A2PM	19	T	**K**	**P**	I	G	M	**R**	D	R	**E**
2		2	**I**	E	**P**	I	G	M	K	D	R	Q
1	TCGA-EK-A2R7	30	T	E	**P**	I	G	M	**R**	D	R	Q
2		21	**I**	E	**P**	I	G	M	K	D	R	**E**
1	TCGA-EK-A2R8	1	T	E	R	I	G	M	K	D	R	Q
2		18	T	E	**P**	**M**	G	M	**R**	D	R	**E**
1	TCGA-EK-A2R9	6	T	E	R	**M**	G	M	**R**	D	R	**E**
2		23	T	E	**P**	I	**D**	M	**R**	D	R	**E**
1	TCGA-EK-A2RA	44	**I**	E	**P**	I	G	M	**R**	D	R	Q
2		45	T	**K**	**P**	I	G	M	K	D	R	**E**
1	TCGA-EK-A2RB	5	T	E	**P**	I	G	**V**	**R**	**N**	**Q**	**E**
2		18	T	E	**P**	**M**	G	M	**R**	D	R	**E**
1	TCGA-EK-A2RC	18	T	E	**P**	**M**	G	M	**R**	D	R	**E**
2		5	T	E	**P**	I	G	**V**	**R**	**N**	**Q**	**E**
1	TCGA-EK-A2RE	38	T	E	**P**	I	G	M	K	D	R	**E**
2		6	T	E	R	**M**	G	M	**R**	D	R	**E**
1	TCGA-EK-A2RJ	23	T	E	**P**	I	**D**	M	**R**	D	R	**E**
2		31	T	E	R	**M**	G	M	K	D	R	**E**
1	TCGA-EK-A2RK	18	T	E	**P**	**M**	G	M	**R**	D	R	**E**
2		18	T	E	**P**	**M**	G	M	**R**	D	R	**E**
1	TCGA-EK-A2RL	45	T	**K**	**P**	I	G	M	K	D	R	**E**
2		46	T	E	R	I	G	M	**R**	D	R	Q
1	TCGA-EK-A2RM	18	T	E	**P**	**M**	G	M	**R**	D	R	**E**
2		18	T	E	**P**	**M**	G	M	**R**	D	R	**E**
1	TCGA-EK-A2RN	18	T	E	**P**	**M**	G	M	**R**	D	R	**E**
2		18	T	E	**P**	**M**	G	M	**R**	D	R	**E**
1	TCGA-EK-A3GK	38	T	E	**P**	I	G	M	K	D	R	**E**
2		14	T	E	R	I	G	M	K	D	R	**E**
1	TCGA-EK-A3GM	19	T	**K**	**P**	I	G	M	**R**	D	R	**E**

2		34	I	E	P	I	G	M	R	D	R	E
1	TCGA-EK-A3GN	38	T	E	P	I	G	M	K	D	R	E
2		47	T	E	R	I	G	V	R	N	Q	E
1	TCGA-EX-A1H5	10	T	E	P	I	G	M	R	D	R	E
2		18	T	E	P	M	G	M	R	D	R	E
1	TCGA-EX-A1H6	38	T	E	P	I	G	M	K	D	R	E
2		32	T	E	P	M	G	M	R	D	R	Q
1	TCGA-EX-A3L1	19	T	K	P	I	G	M	R	D	R	E
2		10	T	E	P	I	G	M	R	D	R	E
1	TCGA-EX-A69L	1	T	E	R	I	G	M	K	D	R	Q
2		18	T	E	P	M	G	M	R	D	R	E
1	TCGA-EX-A69M	18	T	E	P	M	G	M	R	D	R	E
2		18	T	E	P	M	G	M	R	D	R	E
1	TCGA-FU-A23K	44	I	E	P	I	G	M	R	D	R	Q
2		9	T	E	P	M	G	M	K	D	R	E
1	TCGA-FU-A23L	2	I	E	P	I	G	M	K	D	R	Q
2		5	T	E	P	I	G	V	R	N	Q	E
1	TCGA-FU-A2QG	26	T	E	P	I	G	M	R	N	Q	E
2		10	T	E	P	I	G	M	R	D	R	E
1	TCGA-FU-A3HY	18	T	E	P	M	G	M	R	D	R	E
2		18	T	E	P	M	G	M	R	D	R	E
1	TCGA-FU-A3NI	1	T	E	R	I	G	M	K	D	R	Q
2		38	T	E	P	I	G	M	K	D	R	E
1	TCGA-FU-A3TQ	18	T	E	P	M	G	M	R	D	R	E
2		5	T	E	P	I	G	V	R	N	Q	E
1	TCGA-FU-A3WB	4	T	E	P	I	G	V	R	N	R	Q
2		22	T	E	R	I	G	M	K	D	Q	E
1	TCGA-FU-A3YQ	18	T	E	P	M	G	M	R	D	R	E
2		1	T	E	R	I	G	M	K	D	R	Q
1	TCGA-FU-A40J	19	T	K	P	I	G	M	R	D	R	E

2		2	I	E	P	I	G	M	K	D	R	Q
1	TCGA-FU-A57G	6	T	E	R	M	G	M	R	D	R	E
2		38	T	E	P	I	G	M	K	D	R	E
1	TCGA-FU-A5XV	48	T	E	R	I	G	V	R	N	R	E
2		42	T	E	P	I	G	M	K	D	Q	E
1	TCGA-FU-A770	14	T	E	R	I	G	M	K	D	R	E
2		49	T	E	P	I	G	V	R	N	Q	Q
1	TCGA-HG-A2PA	6	T	E	R	M	G	M	R	D	R	E
2		38	T	E	P	I	G	M	K	D	R	E
1	TCGA-HM-A3JJ	18	T	E	P	M	G	M	R	D	R	E
2		10	T	E	P	I	G	M	R	D	R	E
1	TCGA-HM-A3JK	2	I	E	P	I	G	M	K	D	R	Q
2		1	T	E	R	I	G	M	K	D	R	Q
1	TCGA-HM-A4S6	50	T	E	R	I	D	V	R	N	Q	E
2		10	T	E	P	I	G	M	R	D	R	E
1	TCGA-HM-A6W2	14	T	E	R	I	G	M	K	D	R	E
2		1	T	E	R	I	G	M	K	D	R	Q
1	TCGA-IR-A3L7	51	T	E	P	I	G	V	R	D	R	E
2		26	T	E	P	I	G	M	R	N	Q	E
1	TCGA-IR-A3LA	10	T	E	P	I	G	M	R	D	R	E
2		18	T	E	P	M	G	M	R	D	R	E
1	TCGA-IR-A3LC	2	I	E	P	I	G	M	K	D	R	Q
2		18	T	E	P	M	G	M	R	D	R	E
1	TCGA-IR-A3LF	10	T	E	P	I	G	M	R	D	R	E
2		18	T	E	P	M	G	M	R	D	R	E
1	TCGA-IR-A3LH	7	T	E	P	I	G	M	K	D	R	Q
2		6	T	E	R	M	G	M	R	D	R	E
1	TCGA-IR-A3LI	5	T	E	P	I	G	V	R	N	Q	E
2		14	T	E	R	I	G	M	K	D	R	E
1	TCGA-IR-A3LK	5	T	E	P	I	G	V	R	N	Q	E

2		18	T	E	**P**	**M**	G	M	**R**	D	R	**E**
1	TCGA-IR-A3LL	19	T	**K**	**P**	I	G	M	**R**	D	R	**E**
2		10	T	E	**P**	I	G	M	**R**	D	R	**E**
1	TCGA-JW-A5VG	1	T	E	R	I	G	M	K	D	R	Q
2		8	T	E	R	I	G	M	**R**	D	R	**E**
1	TCGA-JW-A5VH	1	T	E	R	I	G	M	K	D	R	Q
2		18	T	E	**P**	**M**	G	M	**R**	D	R	**E**
1	TCGA-JW-A5VI	2	**I**	E	**P**	I	G	M	K	D	R	Q
2		1	T	E	R	I	G	M	K	D	R	Q
1	TCGA-JW-A5VJ	7	T	E	**P**	I	G	M	K	D	R	Q
2		6	T	E	R	**M**	G	M	**R**	D	R	**E**
1	TCGA-JW-A5VK	2	**I**	E	**P**	I	G	M	K	D	R	Q
2		14	T	E	R	I	G	M	K	D	R	**E**
1	TCGA-JW-A5VL	34	**I**	E	**P**	I	G	M	**R**	D	R	**E**
2		2	**I**	E	**P**	I	G	M	K	D	R	Q
1	TCGA-JW-A69B	1	T	E	R	I	G	M	K	D	R	Q
2		1	T	E	R	I	G	M	K	D	R	Q
1	TCGA-JX-A3PZ	23	T	E	**P**	I	**D**	M	**R**	D	R	**E**
2		6	T	E	R	**M**	G	M	**R**	D	R	**E**
1	TCGA-JX-A3Q0	18	T	E	**P**	**M**	G	M	**R**	D	R	**E**
2		18	T	E	**P**	**M**	G	M	**R**	D	R	**E**
1	TCGA-JX-A3Q8	14	T	E	R	I	G	M	K	D	R	**E**
2		32	T	E	**P**	**M**	G	M	**R**	D	R	Q
1	TCGA-JX-A5QV	2	**I**	E	**P**	I	G	M	K	D	R	Q
2		1	T	E	R	I	G	M	K	D	R	Q
1	TCGA-LP-A4AU	18	T	E	**P**	**M**	G	M	**R**	D	R	**E**
2		18	T	E	**P**	**M**	G	M	**R**	D	R	**E**
1	TCGA-LP-A4AV	38	T	E	**P**	I	G	M	K	D	R	**E**
2		40	T	E	R	**M**	G	M	**R**	D	R	Q
1	TCGA-LP-A4AW	32	T	E	**P**	**M**	G	M	**R**	D	R	Q

2		14	T	E	R	I	G	M	K	D	R	**E**
1	TCGA-LP-A4AX	19	T	**K**	**P**	I	G	M	**R**	D	R	**E**
2		1	T	E	R	I	G	M	K	D	R	Q
1	TCGA-LP-A5U2	32	T	E	**P**	**M**	G	M	**R**	D	R	Q
2		14	T	E	R	I	G	M	K	D	R	**E**
1	TCGA-LP-A5U3	1	T	E	R	I	G	M	K	D	R	Q
2		18	T	E	**P**	**M**	G	M	**R**	D	R	**E**
1	TCGA-LP-A7HU	14	T	E	R	I	G	M	K	D	R	**E**
2		52	T	**K**	**P**	I	G	M	**R**	D	R	Q
1	TCGA-MU-A51Y	14	T	E	R	I	G	M	K	D	R	**E**
2		18	T	E	**P**	**M**	G	M	**R**	D	R	**E**
1	TCGA-MU-A5YI	18	T	E	**P**	**M**	G	M	**R**	D	R	**E**
2		5	T	E	**P**	I	G	**V**	**R**	**N**	**Q**	**E**
1	TCGA-MY-A5BD	1	T	E	R	I	G	M	K	D	R	Q
2		1	T	E	R	I	G	M	K	D	R	Q
1	TCGA-MY-A5BE	14	T	E	R	I	G	M	K	D	R	**E**
2		1	T	E	R	I	G	M	K	D	R	Q
1	TCGA-MY-A5BF	14	T	E	R	I	G	M	K	D	R	**E**
2		1	T	E	R	I	G	M	K	D	R	Q
1	TCGA-Q1-A5R1	5	T	E	**P**	I	G	**V**	**R**	**N**	**Q**	**E**
2		19	T	**K**	**P**	I	G	M	**R**	D	R	**E**
1	TCGA-Q1-A6DT	5	T	E	**P**	I	G	**V**	**R**	**N**	**Q**	**E**
2		20	T	E	R	I	**D**	M	**R**	D	R	**E**
1	TCGA-Q1-A6DV	38	T	E	**P**	I	G	M	K	D	R	**E**
2		40	T	E	R	**M**	G	M	**R**	D	R	Q
1	TCGA-Q1-A6DW	20	T	E	R	I	**D**	M	**R**	D	R	**E**
2		5	T	E	**P**	I	G	**V**	**R**	**N**	**Q**	**E**
1	TCGA-Q1-A73O	19	T	**K**	**P**	I	G	M	**R**	D	R	**E**
2		14	T	E	R	I	G	M	K	D	R	**E**

1	TCGA-Q1-A73P	2	**I**	E	**P**	I	G	M	K	D	R	Q
2		10	T	E	**P**	I	G	M	**R**	D	R	**E**
1	TCGA-Q1-A73Q	9	T	E	**P**	**M**	G	M	K	D	R	**E**
2		46	T	E	R	I	G	M	**R**	D	R	Q
1	TCGA-Q1-A73R	21	**I**	E	**P**	I	G	M	K	D	R	**E**
2		32	T	E	**P**	**M**	G	M	**R**	D	R	Q
1	TCGA-Q1-A73S	43	T	E	R	I	G	**V**	**R**	**N**	R	Q
2		42	T	E	**P**	I	G	M	K	D	**Q**	**E**
1	TCGA-R2-A69V	1	T	E	R	I	G	M	K	D	R	Q
2		14	T	E	R	I	G	M	K	D	R	**E**
1	TCGA-RA-A741	17	T	E	R	I	**D**	M	**R**	D	R	Q
2		14	T	E	R	I	G	M	K	D	R	**E**

Each patient has two haplotypes that are composed by the combination of SNPs: rs72773968 (T12I), rs3734016 (p.Glu56Lys), rs26653 (p.Arg127Pro), rs26618 (p.Ile276Met), rs27895 (p.Gly346Asp), rs2287987 (p.Met349Val), rs30187 (p.Lys528Arg), rs10050860 (p.Asp575Asn), rs17482078 (p.Arg725Gln) and rs27044 (p.Gln730Glu). In red, the amino acids that are altered in relation to the wild-type sequence of *ERAP1* due to the presence of the SNP in the *locus* in question.

Table S7. Kaplan-Meier survival analysis with Log-Rank test for ERAP1 variants and clinicopathological characteristics in TCGA cervical cancer patients.

Variant		OS p-value	DSS p-value
rs3734016	p.Glu56Lys	0.35	0.14
rs26653	p.Arg127Pro	0.19	0.14
rs72773968	p.Thr12Ile	0.95	0.96
rs26618	p.Ile276Met	0.85	0.51
rs27895	p.Gly346Asp	0.29	0.12
rs2287987	p.Met349Val	0.028*	0.009*
rs30187	p.Lys528Arg	0.056*	0.13
rs10050860	p.Asp575Asn	0.91	0.57
rs17482078	p.Arg725Gln	0.87	0.59
rs27044	p.Gln730Glu	0.42	0.48
**Clinicopathological characteristics**			
FIGO staging		0,042*	0,12
Age		0.39	0,8

Table S8. Kaplan-Meier survival analysis with Log-Rank test for ERAP1 variants and clinicopathological characteristics in Brazilian cervical cancer patients

Variant		OS p-value	DSS p-value
rs72773968	p.Thr12Ile	0,66	0.78
rs2287987	p.Met349Val	0.59	0.42
rs30187	p.Lys528Arg	0.55	0.58
rs10050860	p.Asp575Asn	0.65	0.90
rs17482078	p.Arg725Gln	0.47	0.44
rs27044	p.Gln730Glu	0.38	0.31
**Clinicopathological characteristics**			
FIGO staging		0.24	0.3
Age		0.98	0.63

Table S9. Cox regression analysis for overall survival (OS) in Brazilian cervical cancer patients considering *ERAP1* variants and clinicopathological variables. The table presents the hazard ratios (HRs), 95% confidence intervals (CIs), and p-values for each variant and variable in both the initial and final models. Statistically significant results are marked with an asterisk (*), indicating a p-value < 0.05.

Variable			Overall survival		
			**HR**	**95% CI**	***P-value***
rs72773968	p.Thr12Ile	Homozygous/Heterozygous	1.6071	0.1876 - 13.77	0.665
rs2287987	p.Met349Val	Homozygous/Heterozygous	1.6055	0.2939 - 8.77	0.585
rs30187	p.Lys528Arg	Homozygous/Heterozygous	2.020	0.2358 - 17.3	0.521
rs10050860	p.Asp575Asn	Homozygous/Heterozygous	1.3666	0.25 - 7.471	0.719
rs17482078	p.Arg725Gln	Homozygous/Heterozygous	1.8577	0.34 - 10.15	0.475
rs27044	p.Gln730Glu	Homozygous/Heterozygous	7.750e+07	0 - Inf	0.999
FIGO stage		II	3.928e+07	0 - Inf	0.999
		III_IV	1.411e+08	0 - Inf	0.998
Age		<49y vs. >49y	1.02223	0.2062 - 5.068	0.979

Table S10. Cox regression analysis for disease-specific survival (DSS) in Brazilian cervical cancer patients considering *ERAP1* variants and clinicopathological variables. The table presents the hazard ratios (HRs), 95% confidence intervals (CIs), and p-values for each variant and variable in both the initial and final models. Statistically significant results are marked with an asterisk (*), indicating a p-value < 0.05.

Variable			Disease specific survival		
			**HR**	**95% CI**	***P-value***
rs72773968	p.Thr12Ile	Homozygous/Heterozygous	0.8177	0.1884 - 3.549	0.788
rs2287987	p.Met349Val	Homozygous/Heterozygous	0.5938	0.1991 - 1771	0.35
rs30187	p.Lys528Arg	Homozygous/Heterozygous	0.98502	0.3783 - 2.565	0.975
rs10050860	p.Asp575Asn	Homozygous/Heterozygous	0.91492	0.3557 - 2.353	0.854
rs17482078	p.Arg725Gln	Homozygous/Heterozygous	0.5811	0.1707 - 1.979	0.385
rs27044	p.Gln730Glu	Homozygous/Heterozygous	1.03363	0.2396 - 4.459	0.965
FIGO stage		II	2.3859	0.2907 - 19.58	0.418
		III_IV	3.7163	0.4854 - 28.45	0.206
Age		<49y vs. >49y	0.8098	0.341 - 1.923	0.633

S11 Table. Association analysis between HLA I genotypes and FIGO staging among the TCGA-CESC dataset.

Alleles	Genetic model								
	Allelic			Dominant			Recessive		
	Frequency	P-value	OR (95% CI)	Frequency	P-value	OR (95% CI)	Frequency	P-value	OR (95% CI)
**A*01:01**	0.1233	0.632	0.7800 (0.3732-1.6300)	0.2397	0.4429	0.6722 (0.2996-1.5081)	---	---	---
**A*02:01**	0.2123	0.7062	0.8556 (0.4772-1.5338)	0.363	0.7693	0.8458 (0.4207-1.7003)	0.0616	0.9729	0.7925 (0.1900-3.3046)
**A*02:03**	0.0103	0.6767	3.2545 (0.2917-36.3164)	0.0205	0.6752	3.2963 (0.2919-37.2254)	---	---	---
**A*02:05**	0.0103	0.6767	0.8018 (0.0719-8.9467)	0.0205	0.6752	0.8000 (0.0709-9.0330)	---	---	---
**A*02:06**	0.0171	0.6983	1.0727 (0.1764-6.5220)	0.0342	0.6958	1.0741 (0.1738-6.6367)	---	---	---
**A*03:01**	0.1301	0.7005	0.8141 (0.3978-1.6660)	0.2192	0.7502	0.8032 (0.3536-1.8245)	0.0411	0.8648	0.7963 (0.1410-4.4970)
**A*11:01**	0.0582	0.9916	1.1333 (0.4186-3.0686)	0.1164	0.9913	1.1429 (0.4083-3.1989)	---	---	---
**A*23:01**	0.0205	0.8662	1.6239 (0.3220-8.1893)	0.0411	0.8648	1.6415 (0.3196-8.4316)	---	---	---
**A*24:02**	0.1062	0.5295	1.3697 (0.6467-2.9010)	0.1849	0.6161	1.3636 (0.5855-3.1759)	0.0274	0.9715	1.6296 (0.2230-11.9105)
**A*24:07**	0.0103	0.6767	0.8018 (0.0719-8.9467)	0.0205	0.6752	0.8000 (0.0709-9.0330)	---	---	---
**A*26:01**	0.0205	0.8662	0.8000 (0.1441-4.4411)	0.0411	0.8648	0.7963 (0.1410-4.4970)	---	---	---
**A*29:02**	0.0342	0.8242	1.0741 (0.2963-3.8931)	0.0685	0.8211	1.0769 (0.2901-3.9976)	---	---	---
**A*30:01**	0.0479	0.9416	1.2170 (0.4109-3.6046)	0.089	0.7589	1.4229 (0.4526-4.4735)	---	---	---
**A*30:02**	0.0103	0.6767	0.8018 (0.0719-8.9467)	0.0205	0.6752	0.8000 (0.0709-9.0330)	---	---	---
**A*31:01**	0.0342	0.8242	0.6802 (0.1722-2.6865)	0.0685	0.8211	0.6712 (0.1662-2.7101)	---	---	---
**A*32:01**	0.0171	0.6983	1.0727 (0.1764-6.5220)	0.0342	0.6958	1.0741 (0.1738-6.6367)	---	---	---
**A*33:03**	0.0171	0.6983	1.0727 (0.1764-6.5220)	0.0342	0.6958	1.0741 (0.1738-6.6367)	---	---	---
**A*34:01**	---	---	---	0.0137	0.6957	1.6182 (0.0992-26.4029)	---	---	---
**A*68:01**	0.0308	0.4656	2.0561 (0.5402-7.8254)	0.0616	0.4583	2.1078 (0.5412-8.2099)	---	---	---
**A*68:02**	0.0171	0.5891	2.4495 (0.4029-14.8937)	0.0342	0.5858	2.4906 (0.4030-15.3928)	---	---	---
**B*07:02**	0.1233	0.9102	1.0260 (0.5014-2.0992)	0.2329	0.8534	1.1667 (0.5333-2.5523)	---	---	---
**B*08:01**	0.0959	0.9219	1.0443 (0.4702-2.3194)	0.1849	0.9497	1.1306 (0.4820-2.6515)	---	---	---

**B*13:02**	0.0377	0.8591	1.3551 (0.4037-4.5491)	0.0685	0.6544	1.6667 (0.4600-6.0384)	---	---	---
**B*14:02**	0.0205	0.8662	1.6239 (0.3220-8.1893)	0.0411	0.8648	1.6415 (0.3196-8.4316)	---	---	---
**B*15:01**	0.0171	0.6983	1.0727 (0.1764-6.5220)	0.0342	0.6958	1.0741 (0.1738-6.6367)	---	---	---
**B*15:02**	---	---	---	0.0137	0.6957	1.6182 (0.0992-26.4029)	---	---	---
**B*15:10**	0.0103	0.6767	3.2545 (0.2917-36.3164)	0.0205	0.6752	3.2963 (0.2919-37.2254)	---	---	---
**B*18:01**	0.024	0.8843	1.2110 (0.2659-5.5144)	0.0479	0.8829	1.2170 (0.2620-5.6518)	---	---	---
**B*27:05**	0.0377	0.2772	0.3455 (0.0733-1.6289)	0.0753	0.2676	0.3333 (0.0693-1.6029)	---	---	---
**B*35:01**	0.0479	0.5244	1.6476 (0.5621-4.8292)	0.0959	0.5136	1.6939 (0.5607-5.1171)	---	---	---
**B*35:03**	---	---	---	0.0137	0.6957	1.6182 (0.0992-26.4029)	---	---	---
**B*35:05**	0.0103	0.6767	0.8018 (0.0719-8.9467)	0.0205	0.6752	0.8000 (0.0709-9.0330)	---	---	---
**B*35:08**	0.0103	0.6767	0.8018 (0.0719-8.9467)	0.0205	0.6752	0.8000 (0.0709-9.0330)	---	---	---
**B*40:01**	0.0377	0.8591	0.9153 (0.2618-3.2006)	0.0685	0.8211	0.6712 (0.1662-2.7101)	---	---	---
**B*40:02**	0.0171	0.5891	2.4495 (0.4029-14.8937)	0.0342	0.5858	2.4906 (0.4030-15.3928)	---	---	---
**B*44:02**	0.0788	0.8856	1.0361 (0.4329-2.4798)	0.1575	0.8805	1.0395 (0.4172-2.5902)	---	---	---
**B*44:03**	0.0548	0.8478	0.9623 (0.3398-2.7246)	0.1096	0.8432	0.9600 (0.3286-2.8045)	---	---	---
**B*46:01**	0.0137	0.9717	0.5315 (0.0546-5.1739)	0.0274	0.9715	0.5273 (0.0535-5.1977)	---	---	---
**B*49:01**	0.0137	0.9717	0.5315 (0.0546-5.1739)	0.0274	0.9715	0.5273 (0.0535-5.1977)	---	---	---
**B*50:01**	0.0171	0.6983	1.0727 (0.1764-6.5220)	0.0342	0.6958	1.0741 (0.1738-6.6367)	---	---	---
**B*51:01**	0.0377	0.8591	0.9153 (0.2618-3.2006)	0.0753	0.8563	0.9121 (0.2545-3.2689)	---	---	---
**B*52:01**	0.0137	0.9717	0.5315 (0.0546-5.1739)	0.0274	0.9715	0.5273 (0.0535-5.1977)	---	---	---
**B*53:01**	0.0137	0.9717	1.6182 (0.2247-11.6542)	0.0274	0.9715	1.6296 (0.2230-11.9105)	---	---	---
**B*55:01**	0.0205	0.8662	1.6239 (0.3220-8.1893)	0.0411	0.8648	1.6415 (0.3196-8.4316)	---	---	---
**B*57:01**	0.0342	0.2707	2.4906 (0.6870-9.0289)	0.0616	0.4583	2.1078 (0.5412-8.2099)	---	---	---
**B*58:01**	0.0137	0.3173	4.9266 (0.5061-47.9595)	0.0274	0.314	5.0377 (0.5109-49.6787)	---	---	---
**B*58:02**	---	---	---	0.0137	0.6957	1.6182 (0.0992-26.4029)	---	---	---
**B*82:01**	---	---	---	0.0137	0.6957	1.6182 (0.0992-26.4029)	---	---	---
**C*01:02**	0.0514	0.4944	0.5690 (0.1767-1.8326)	0.1027	0.4823	0.5524 (0.1669-1.8282)	---	---	---
**C*02:02**	0.0308	0.5074	0.4494 (0.0917-2.2026)	0.0616	0.5005	0.4392 (0.0879-2.1935)	---	---	---

**C*03:02**	0.0103	0.6767	3.2545 (0.2917-36.3164)	0.0205	0.6752	3.2963 (0.2919-37.2254)	---	---	---
**C*03:03**	0.0445	0.7766	1.0047 (0.3203-3.1512)	0.089	0.7713	1.0049 (0.3116-3.2403)	---	---	---
**C*03:04**	0.0479	0.9416	1.2170 (0.4109-3.6046)	0.0959	0.94	1.2300 (0.4032-3.7525)	---	---	---
**C*04:01**	0.1062	0.8788	1.0168 (0.4734-2.1843)	0.2123	0.8709	1.0191 (0.4512-2.3017)	---	---	---
**C*05:01**	0.0719	0.8357	1.2233 (0.4981-3.0041)	0.137	0.9325	1.0833 (0.4131-2.8412)	---	---	---
**C*06:02**	0.1027	0.4295	1.4643 (0.6850-3.1301)	0.1781	0.2609	1.7907 (0.7619-4.2087)	0.0274	0.9715	0.5273 (0.0535-5.1977)
**C*07:01**	0.1301	0.7408	1.1970 (0.5991-2.3915)	0.2397	0.6681	1.2805 (0.5914-2.7725)	0.0205	0.6752	0.8000 (0.0709-9.0330)
**C*07:02**	0.1575	0.9621	0.9318 (0.4857-1.7873)	0.3014	0.8889	1.0171 (0.4919-2.1031)	---	---	---
**C*07:04**	0.024	0.8843	0.6364 (0.1213-3.3372)	0.0479	0.8829	0.6296 (0.1179-3.3613)	---	---	---
**C*07:06**	---	---	---	0.0137	0.6957	1.6182 (0.0992-26.4029)	---	---	---
**C*08:01**	0.0103	0.6767	0.8018 (0.0719-8.9467)	0.0205	0.6752	0.8000 (0.0709-9.0330)	---	---	---
**C*08:02**	0.024	0.5213	2.1852 (0.4798-9.9511)	0.0479	0.5161	2.2308 (0.4802-10.3630)	---	---	---
**C*12:03**	0.0274	0.7504	0.9633 (0.2257-4.1117)	0.0548	0.7469	0.9623 (0.2208-4.1931)	---	---	---
**C*14:02**	0.0103	0.6767	0.8018 (0.0719-8.9467)	0.0205	0.6752	0.8000 (0.0709-9.0330)	---	---	---
**C*15:02**	0.0274	0.7504	0.9633 (0.2257-4.1117)	0.0548	0.7469	0.9623 (0.2208-4.1931)	---	---	---
**C*16:01**	0.0377	0.4182	1.9811 (0.5901-6.6511)	0.0753	0.4088	2.0400 (0.5920-7.0296)	---	---	---
**C*17:01**	0.0205	0.4966	0.3153 (0.0364-2.7347)	0.0411	0.492	0.3091 (0.0352-2.7171)	---	---	---
